# Human and planetary health implications of negative emissions technologies

**DOI:** 10.1038/s41467-022-30136-7

**Published:** 2022-05-09

**Authors:** Selene Cobo, Ángel Galán-Martín, Victor Tulus, Mark A. J. Huijbregts, Gonzalo Guillén-Gosálbez

**Affiliations:** 1 Department of Chemistry and Applied Biosciences, Institute for Chemical and Bioengineering, ETH Zürich, Zürich, Switzerland; 2 Department of Chemical, Environmental and Materials Engineering, Universidad de Jaén, Jaén, Spain; 3 Center for Advanced Studies in Earth Sciences, Energy and Environment, Universidad de Jaén, Jaén, Spain; 4 Department of Environmental Science, Radboud Institute for Biological and Environmental Sciences, Radboud University, Nijmegen, The Netherlands

**Keywords:** Environmental impact, Risk factors

## Abstract

Meeting the 1.5 °C target may require removing up to 1,000 Gtonne CO_2_ by 2100 with Negative Emissions Technologies (NETs). We evaluate the impacts of Direct Air Capture and Bioenergy with Carbon Capture and Storage (DACCS and BECCS), finding that removing 5.9 Gtonne/year CO_2_ can prevent <9·10^2^ disability-adjusted life years per million people annually, relative to a baseline without NETs. Avoiding this health burden—similar to that of Parkinson’s—can save substantial externalities (≤148 US$/tonne CO_2_), comparable to the NETs levelized costs. The health co-benefits of BECCS, dependent on the biomass source, can exceed those of DACCS. Although both NETs can help to operate within the climate change and ocean acidification planetary boundaries, they may lead to trade-offs between Earth-system processes. Only DACCS can avert damage to the biosphere integrity without challenging other biophysical limits (impacts ≤2% of the safe operating space). The quantified NETs co-benefits can incentivize their adoption.

## Abstract (web summary)

This paper assesses the co-benefits and side-effects of DACCS and BECCS for human health and the planet. The health burden avoided by removing 5.9 Gtonne/yr CO2 is substantial, but only DACCS has a low impact on the Earth system.

## Introduction

Limiting global warming to 1.5 °C above pre-industrial levels with no or limited overshoot will require removing up to ~1000 Gtonne CO_2_ from the atmosphere by 2100 (median estimate: 584 Gtonne)^1^. Nonetheless, it is still unclear whether the benefits of deploying Negative Emissions Technologies (NETs) at large scale would offset their potential damaging effects on humans and the planet.

Prior studies on Direct Air Carbon Capture and Storage (DACCS) and Bioenergy with Carbon Capture and Storage (BECCS)—two of the most prominent NETs—^2,3^, primarily focused on analyzing their costs^4–6^ and CO_2_ removal (CDR) potentials^7–14^. In contrast, their side-effects and co-benefits beyond global warming have often been overlooked. Some studies have quantified the environmental impacts of DACCS and BECCS, but their results are hard to interpret from an absolute sustainability viewpoint^15–19^. Only recently, the impacts of BECCS were assessed against the Earth’s biophysical limits^20,21^, i.e., the Planetary Boundaries (PBs) within which humanity could safely operate^22^.

Notwithstanding these scarce global studies, a comprehensive analysis of the implications of DACCS and BECCS embracing simultaneously human and planetary health is lacking. Filling this knowledge gap is critical to uncover the co-benefits of CDR and minimize the potential collateral damage of combating climate change—especially considering that environmental trade-offs frequently arise in the energy sector^23,24^, which is deeply intertwined with NETs^25^.

Here we quantify the human health impacts of DACCS and BECCS in terms of Disability-Adjusted Life Years (DALYs), alongside their planetary footprint on seven Earth-system processes key to maintaining the Earth’s stability. We found that DACCS and BECCS could preserve a substantial number of years of healthy life,—on the order of the healthy life years lost annually due to Parkinson’s disease—, with Africa and Asia benefiting the most from CDR because of the avoided risk of undernutrition and malaria. Nevertheless, these NETs could also generate detrimental health impacts associated with pollutant emissions and water consumption, chiefly at the regional level. Both NETs could avert the future climate change and ocean acidification impacts of past carbon emissions. However, the large-scale deployment of BECCS could exert substantial pressure on the terrestrial biosphere, nitrogen biogeochemical flows and freshwater use Earth-system processes. In contrast, DACCS emerges as environmentally superior due to its lower planetary impact and its ability to prevent adverse side-effects on the terrestrial biosphere.

The long-term global co-benefits of CDR on human and planetary health, quantified here for the first time, could act as solid incentives to promote NETs, helping to accelerate the climate change mitigation agenda.

## Results

### Scenario definition

We studied 16 scenarios removing 5.9 net Gtonne/year CO_2_ between 2030 and 2100. This corresponds to the average annual CDR rate in climate change mitigation scenario SSP2-1.9 (marker scenario, model MESSAGE-GLOBIOM)^26^, excluding CDR in the agriculture, forestry and other land-use sector. Hence, the underlying assumptions of our scenarios are those adopted in the SSP2-1.9 marker scenario^27,28^, which limits the temperature increase to 1.3 °C above pre-industrial levels by 2100 and is based on Shared Socioeconomic Pathway 2 (SSP2). The middle-of-the-road narrative of SSP2 is consistent with development trends following historical patterns, persistent income inequality and moderate global population growth. This pathway presents slow progress toward achieving the Sustainable Development Goals (SDGs) and overall reductions in resource and energy use, which are not sufficient to halt environmental degradation^29^.

**Fig. 1:**
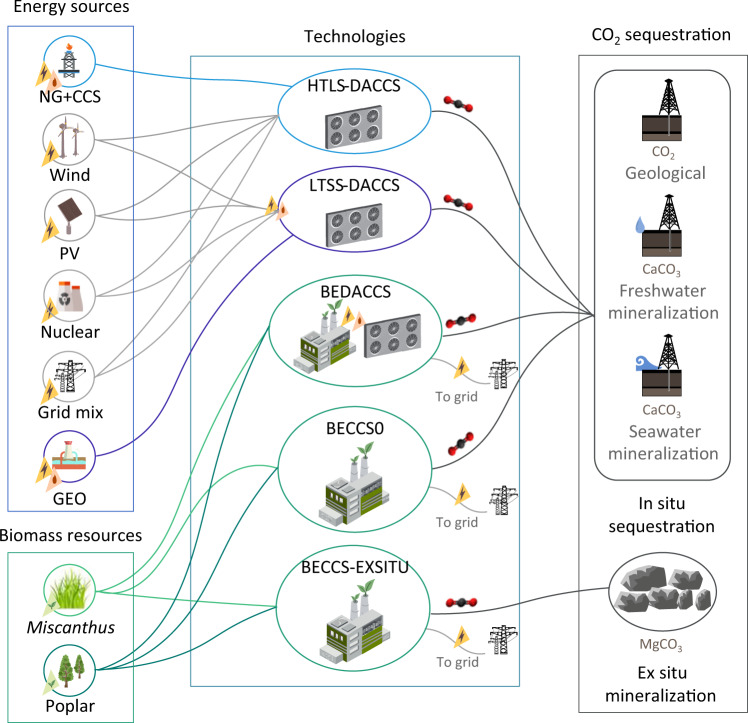
Overview of the assessed NETs scenarios. High-Temperature Liquid Sorbent (HTLS) and Low-Temperature Solid Sorbent (LTSS) Direct Air Carbon Capture and Storage (DACCS)—powered by natural gas with carbon capture and storage (NG+CCS), wind, solar photovoltaic (PV), nuclear, geothermal (GEO), or the global electricity mix—, the basic Bioenergy with Carbon Capture and storage (BECCS) scenarios (BECCS0) deploying *Miscanthus* or poplar, the hybrid BEDACCS configurations integrating BECCS0 and LTSS-DACCS, and the BECCS scenarios where CO_2_ is mineralized ex situ (BECCS-EXSITU). The CO_2_ in situ sequestration options include geological sequestration at high pressure and mineral carbonation with freshwater or seawater. The set of scenarios generating bioenergy include BECCS0, BEDACCS and BECCS-EXSITU, all referred to as BECCS.

The 16 modeled scenarios differ in the deployed NET, the energy and biomass sources, and the CO_2_ storage configurations. We modeled ten DACCS scenarios, four BECCS scenarios, and two hybrid scenarios combining DACCS and BECCS (Fig. 1). We compare these scenarios to a baseline without NETs—otherwise identical to the SSP2-1.9 marker scenario—, which would lead to a mean rise in global temperatures of 1.5 °C with respect to pre-industrial levels (see Temperature in the baseline, in Methods). To facilitate the assessment of our scenarios against the baseline, we define scenario 0, where 5.9 Gtonne/year CO_2_—the difference in net CO_2_ emissions between the baseline and the SSP2-1.9 marker scenario—are emitted.

Concerning the DACCS scenarios, we evaluate High-Temperature Liquid Sorbent (HTLS-DACCS) and Low-Temperature Solid Sorbent (LTSS-DACCS) technologies powered by various energy sources: geothermal (GEO), onshore wind, solar photovoltaic (PV), nuclear, natural gas with carbon capture and storage (NG + CCS), or the global electricity mix deployed between 2030 and 2100, consistent with the SSP2-1.9 marker scenario. Heat is supplied by NG+CCS in the HTLS-DACCS scenarios, whereas either the excess heat from geothermal facilities^30^ or heat pumps are used in the LTSS-DACCS scenarios.

The BECCS scenarios generate electricity from biomass combustion—displacing the global electricity mix of the SSP2-1.9 marker scenario in the period 2030–2100—, and use monoethanolamine to separate CO_2_ from the flue gases^31^. Unlike hydrogen-BECCS, the assessed BECCS systems rely on existing infrastructure for energy distribution and use^32^, and show greater sequestration potential than biofuel-BECCS^13^. In scenario BECCS0-MISC, the boiler is fed with *Miscanthus* grown without irrigation in areas previously classified as grasslands. Scenario BECCS0-POP considers the cultivation of poplar, which requires irrigation. We assume that the land-use change (LUC) from grassland to poplar plantation leads to soil carbon emissions, whereas introducing *Miscanthus* in natural grasslands contributes to soil carbon sequestration^33^.

In the two hybrid scenarios (BEDACCS-MISC and BEDACCS-POP, based on BECCS0-MISC and BECCS0-POP, respectively), a fraction of the low-pressure steam generated in the bioenergy process supplies the heat required to regenerate the monoethanolamine solution, as in the BECCS scenarios (Supplementary Fig. 9). The remaining low-pressure steam alongside the electricity generated with high-pressure steam cover the energy needs of the coupled LTSS-DACCS, capturing 66–70% of the sequestered CO_2_ via BECCS, and the rest, through DACCS.

We study four CO_2_ storage options, namely (1) sequestration at high pressures in geological formations, in situ mineral carbonation^34^ using (2) freshwater or (3) seawater, and (4) ex situ mineral carbonation^35,36^. The latter configuration does not apply to the DACCS scenarios because heat pumps cannot supply the required high-temperature heat^37^. Unless otherwise indicated, the results reported for HTLS-DACCS, LTSS-DACCS, BECCS0 and BEDACCS consider the average impacts of storage options 1, 2, and 3, as labeled above. In scenarios BECCS-EXSITU-MISC and BECCS-EXSITU-POP (based on BECCS0-MISC and BECCS0-POP, respectively), electricity and high-pressure steam diverted from the bioenergy processes cover the energy needed for the ex situ mineralization.

### Human health impacts

We start by analyzing the long-term health effects of scenario 0, where NETs are not deployed. Emitting 5.9 Gtonne/year CO_2_ during the considered period would lead to a rise in the global surface temperature of 0.19 °C [0.11–0.26 °C], causing 9·10^2^ DALYs per million people per year, a health burden similar to that of prostate cancer^38^.

**Fig. 2:**
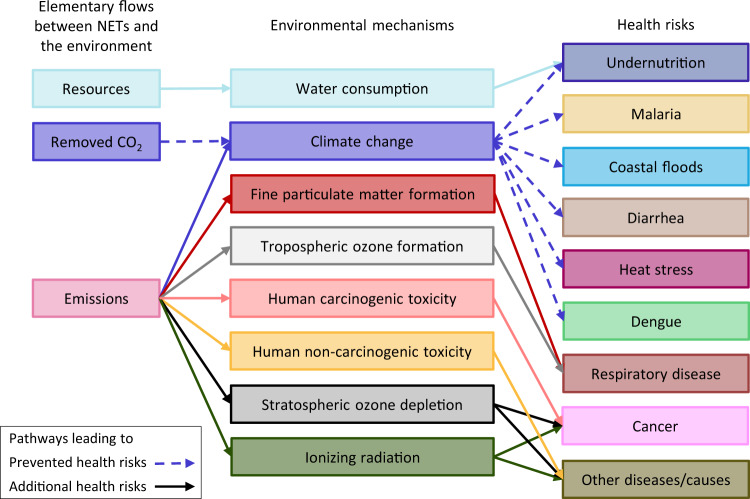
Streamlined human health damage pathways of Negative Emissions Technologies (NETs). These pathways are consistent with the cause-and-effect chains considered by the ReCiPe 2016 method^67^ and Tang et al.^71^.  Dashed and solid arrows lead to prevented and additional health risks, respectively, relative to the baseline.

**Fig. 3:**
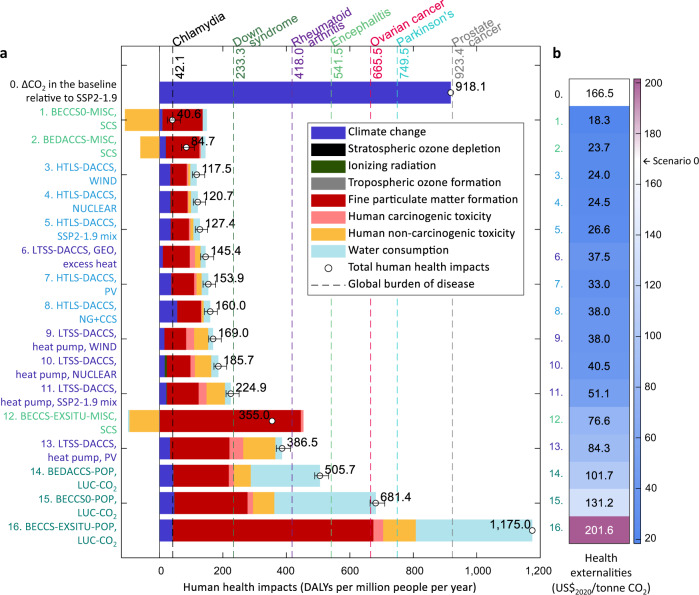
Health impacts. **a** Contribution of environmental mechanisms to the total health impacts, expressed in Disability-Adjusted Life Years (DALYs) per million people per year. Scenarios 1–16 comprise High-Temperature Liquid Sorbent (HTLS) and Low-Temperature Solid Sorbent (LTSS) Direct Air Carbon Capture and Storage (DACCS)—powered by natural gas with carbon capture and storage (NG+CCS), wind, solar photovoltaic (PV), nuclear, geothermal (GEO), or the global electricity mix deployed in the SSP2-1.9 marker scenario without NETs (which limits the increase in radiative forcing to 1.9 W/m^2^ by 2100 and is based on Shared Socioeconomic Pathway 2)—, the basic Bioenergy with Carbon Capture and Storage (BECCS) scenarios (BECCS0) deploying *Miscanthus* (MISC) or poplar (POP)—assuming either Soil Carbon Sequestration (SCS) or land-use change (LUC)—, the hybrid BEDACCS configurations integrating BECCS0 and LTSS-DACCS, and the BECCS scenarios where CO_2_ is mineralized ex situ (BECCS-EXSITU). Scenarios 1–16 are ranked by the total health impacts, scenario 1 is the best. We show the global burden of certain diseases in 2019^38^ for reference. The black bars indicate the health impact range of the scenarios based on the in situ sequestration options, i.e., geological sequestration at high pressure and mineral carbonation with freshwater (upper bound) or seawater (lower bound). **b** Health externalities, expressed in US$_2020_ per gross tonne CO_2_ captured (scenarios 1–16) or emitted (scenario 0).

The long-term health co-benefits of CDR offset the adverse life-cycle health effects associated with freshwater use and pollutant emissions in all the NETs scenarios but one, leading to net health gains between 2·10^2^ and 9·10^2^ DALYs per million people per year with respect to the baseline (health damage pathways of NETs in Fig. 2, scenarios 1–16 ranked according to their health impacts in Fig. 3). Notably, the health impacts prevented in scenario BECCS0-MISC (ranked first) are slightly lower than the global burden of prostate cancer in 2019^38^, while the health savings in the BEDACCS and DACCS scenarios that follow (7 · 10^2^–8 · 10^2^ DALYs per million people per year) are comparable to the annual burden of Parkinson’s disease and higher than that of ovarian cancer^38^.

In the BECCS scenarios, replacing electricity from the global mix with the generated bioelectricity averts additional harmful health effects. The performance of BECCS slightly worsens when integrating it with DACCS—because less electricity is exported to the grid—, and it substantially drops when poplar is the biomass source, mostly due to the water used for irrigation. The health benefits of displacing the grid electricity play an important role in the BECCS scenarios; without the electricity credits, the health impacts that BECCS0-MISC and BECCS0-POP avoid with respect to the baseline would drop by 9% and 26%, respectively (Supplementary Fig. 1).

HTLS-DACCS tends to outperform LTSS-DACCS due to its lower electricity demand, with HTLS-DACCS powered by wind and nuclear energy—both of which attain the lowest emissions of fine particulate matter—ranked third and fourth. The utilization of excess geothermal heat endows LTSS-DACCS with an advantage over the other LTSS-DACCS configurations, while the worst-performing DACCS scenario is LTSS-DACCS deploying PV energy, mainly because of the formation of fine particulate matter associated with the energy required to produce the PV panels. Regarding the sequestration processes, ex situ mineralization is the most damaging storage option in terms of human health, whereas in situ mineralization with seawater minimizes health impacts because of its lower electricity and freshwater requirements (Supplementary Fig. 3).

Focusing on the impact contributors, fine particulate matter formation is the main driver (>44%) of regional health effects—i.e., those affecting the regions where NETs operate, which we did not specify in this analysis—in all scenarios except for BECCS0-POP and BEDACCS-POP. In these two scenarios, the freshwater used for biomass irrigation is the most significant contributor (50% and 47%, respectively) to the regional health impacts (breakdown in Supplementary Fig. 5). Particulate matter is mainly linked to the energy input in the DACCS scenarios, excluding LTSS-DACCS powered by wind and nuclear, where most of the fine particulate matter is associated with the energy consumed in the production of the polyethylenimine that composes the adsorbent. Particulate matter is primarily generated in the biomass combustion in the BECCS0-MISC and BEDACCS-MISC configurations, and in the mining operations related to the ex situ mineralization in the BECCS-EXSITU scenarios. These results suggest that the NETs location could be key to minimizing their detrimental health effects. Notably, DACCS should be prioritized in regions with high renewables or nuclear energy availability. In contrast, BECCS based on irrigated energy crops should be avoided in areas suffering from water scarcity.

Concerning the regional toxicity impacts, HTLS-DACCS outperforms LTSS-DACCS owing to its lower energy consumption. In the BECCS scenarios relying on poplar, the leaching of heavy metals—which mainly occurs in the biomass plantation and the landfill where the fly ashes are disposed of—is responsible for most of the toxicity impacts. Conversely, the BECCS scenarios deploying *Miscanthus* avoid toxicity impacts due to the ability of biomass to retain metals from the soil. Finally, the regional health effects of ozone formation and the global exposure to the ozone-depleting substances and radionuclides embodied in the NETs supply chains are negligible in all the scenarios.

To further contextualize the impacts of NETs, we quantify their health externalities—i.e., their health impacts expressed in monetary terms—in Fig. 3b, where externalities are expressed per gross tonne CO_2_ captured (scenarios 1–16) or emitted (scenario 0). Fifteen scenarios would incur monetized health benefits relative to the baseline, ranging from 35 to 148 US$/tonne CO_2_ (health externalities for the in situ sequestration configurations and additional externalities in Supplementary Figs. 7, 8). The substantial hidden benefits of NETs, often omitted in their economic evaluation and comparable to the levelized CO_2_ costs of scaled-up combustion-BECCS (134–188 US$/tonne)^31^ and HTLS-DACCS (121–249 US$/tonne)^4^, would make these technologies more affordable than initially thought.

**Fig. 4:**
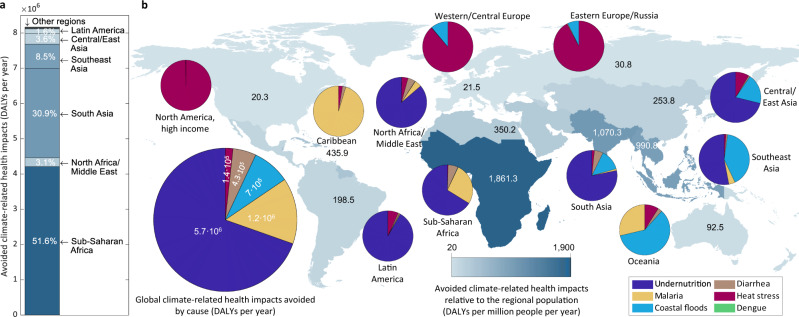
Climate-related health effects avoided with respect to the baseline in the High-Temperature Liquid Sorbent Direct Air Carbon Capture and Storage (HTLS-DACCS) scenario based on wind energy and CO_2_ mineralization with seawater. **a** Geographical distribution of the prevented climate-related health impacts, expressed in Disability-Adjusted Life Years (DALYs) per year. **b** Prevented climate-related health impacts relative to the size of the regional population (DALYs per million people per year) and distribution of the avoided health impacts by cause (DALYs per year). The distribution of the averted climate-related health impacts by region and cause remains constant across the studied scenarios despite the change in the impact magnitude.

We next study the regional and causal distribution of the climate-sensitive health impacts averted in a representative DACCS scenario (HTLS-DACCS deploying wind energy and CO_2_ mineralization with seawater, ranked third in Fig. 3a) with respect to the baseline. This analysis reveals significant disparities across regions, with 98% of the health benefits realized in Africa and Asia, and over half of them in Sub-Saharan Africa (Fig. 4a).

The breakdown of the avoided climate-sensitive DALYs (Fig. 4b) shows that 70% of the human health co-benefits of NETs arise from preventing the climate change impacts on crop productivity, which lead to undernutrition^39^ in Africa, Asia and Latin America. The expected decrease in the incidence of malaria, exacerbated by warm temperatures and rainfall^39^, follows next, representing 15% of the global health savings and mostly benefiting Sub-Saharan Africa. The prevention of coastal floods accounts for 9% of the avoided health impacts. It is noteworthy in Asia (particularly in the East, where 77% of the population lives within 100 km from the coast)^40^ and Oceania. Around 5% of the prevented health impacts stem from the avoided risk of diarrhea, which increases with rising temperatures and little precipitation^39^, and mainly affects Africa and South Asia. The averted impacts of heat stress—more prominent in North America, Europe and Russia—represent 2% of the co-benefits.

In relative terms (considering the population size), Sub-Saharan Africa is the most favored region, with the annual climate-sensitive health impacts averted per million inhabitants almost doubling those in South Asia, which follows next (Fig. 4b). By contrast, North America, Europe, and Russia benefit the least from NETs because they are less sensitive to the health risks intensified by climate change. The health effects prevented in the Caribbean are low in absolute terms but much higher than in the northern areas with respect to their population size, further evidencing the uneven distribution of the health co-benefits across regions. Our life-cycle assessment models preclude a regionalized analysis of the non-climate health impacts. However, the asymmetrical spatial distribution of the prevented climate-related health impacts suggests that the regional health effects of NETs could offset the avoided climate-sensitive health impacts in some locations.

### Impacts on the Earth system

**Fig. 5:**
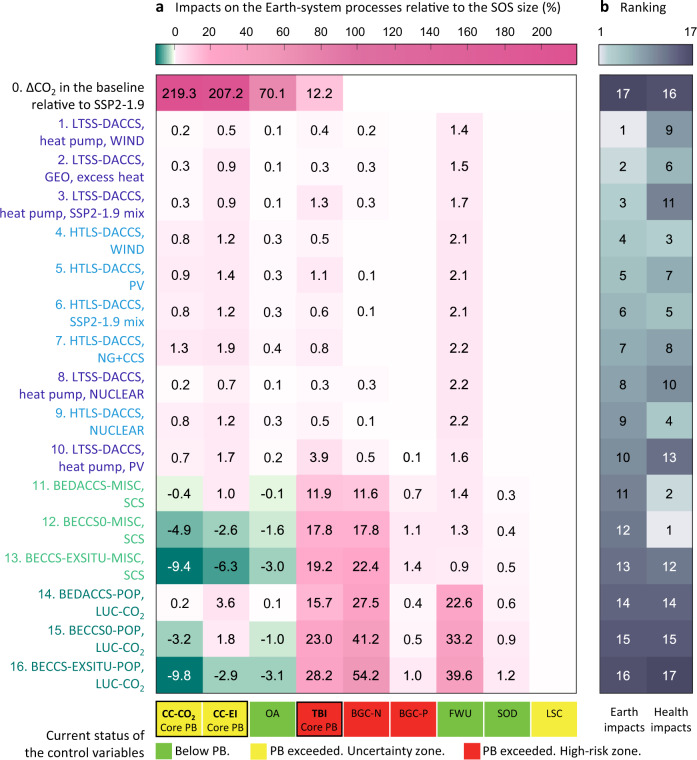
Impacts on the Earth-system processes and ranking of scenarios by impacts on human health and the Earth system. **a** Impacts on Earth-system processes expressed as a percentage of the size of the Safe Operating Space (SOS). The impacts on the following Earth-system processes were assessed: climate change—considering atmospheric CO_2_ concentration (CC-CO_2_) and energy imbalance (CC-EI) as control variables—, ocean acidification (OA), terrestrial biosphere integrity (TBI), global biogeochemical flows—considering the application rate of intentionally fixed reactive N to the agricultural system (BGC-N) and phosphorus flows from freshwater into the ocean (BGC-P) as control variables—, global freshwater use (FWU), stratospheric ozone depletion (SOD), and global land-system change (LSC). Scenarios 1–16 comprise High-Temperature Liquid Sorbent (HTLS) and Low-Temperature Solid Sorbent (LTSS) Direct Air Carbon Capture and Storage (DACCS)—powered by natural gas with carbon capture and storage (NG+CCS), wind, solar photovoltaic (PV), nuclear, geothermal (GEO), or the global electricity mix deployed in the SSP2-1.9 marker scenario (which limits the increase in radiative forcing to 1.9 W/m^2^ by 2100 and is based on Shared Socioeconomic Pathway 2) without NETs—, the basic Bioenergy with Carbon Capture and Storage (BECCS) scenarios (BECCS0) deploying *Miscanthus* (MISC) or poplar (POP)—assuming either Soil Carbon Sequestration (SCS) or land-use change (LUC)—, the hybrid BEDACCS configurations integrating BECCS0 and LTSS-DACCS, and the BECCS scenarios where CO_2_ is mineralized ex situ (BECCS-EXSITU). The values of empty cells range between 0 and 0.05%. We show qualitatively the current level of the control variables for the Planetary Boundaries (PBs) of the studied Earth-system processes below, according to the PB framework^22^. **b** Ranking of scenarios by health impacts and maximum impacts across Earth-system processes relative to the SOS size, scenario 1 is the best.

To quantify the planetary implications of deploying NETs, we assess their impacts on seven critical Earth-system processes relative to the size of the Safe Operating Space (SOS) delimited by the PBs (Fig. 5a, scenarios sorted according to maximum impact across Earth-system processes).

The climate change impacts associated with the CO_2_ emissions of scenario 0—which lead to an increase of 0.19 °C in the global mean temperature by 2100—represent more than twice the climate change SOS. Moreover, the ocean acidification impacts of scenario 0 correspond to 70% of the SOS. Although these impacts are substantial, they are estimated over a 300-year timescale, i.e., they do not occur immediately after the CO_2_ is emitted. Scenario 0 also affects the integrity of the terrestrial biosphere, generating impacts equivalent to 12% of the SOS.

Regarding the NETs scenarios, LTSS-DACCS powered by renewable energy performs best, closely followed by HTLS-DACCS, while the BECCS scenarios show the highest impacts. The studied NETs could avoid impacts equivalent to 204–229% and 70–73% of the climate change and ocean acidification SOSs with respect to the baseline, respectively. The averted impacts are greater in the BECCS scenarios, where bioenergy replaces electricity from the grid, and in the BECCS-EXSITU scenarios, given the avoided impacts related to the byproducts of the ex situ mineralization (see disaggregated contributions in Supplementary Fig. 6).

By contrast, the impacts of BECCS on the terrestrial biosphere exceed those of the baseline by up to 16% of the SOS, whereas DACCS averts impacts equivalent to 8–12% of the biosphere integrity SOS with respect to the baseline. In the BECCS scenarios, the land-use impacts on the terrestrial biosphere outweigh the avoided impacts linked to the removed CO_2_, resulting in net damage to this Earth-system process. The opposite happens in the DACCS scenarios due to their lower land-use requirements.

The use of industrial fertilizers in the BECCS scenarios contributes to further transgressing the biogeochemical flows PBs. While the impacts on the phosphorus flows do not surpass 1% of the SOS in any of the assessed scenarios, the impacts  of the BECCS scenarios deploying *Miscanthus* and poplar represent ≤22% and ≤54% of the nitrogen biogeochemical flow SOS, respectively. Conversely, the impacts of DACCS on the biogeochemical flows are low (≤0.5% of the SOS).

The main unintended impact of the DACCS scenarios stems from their freshwater use, which corresponds to 1–2% of the SOS, with the total freshwater use strongly linked to the sequestration method (Supplementary Fig. 4). The freshwater consumption in the BECCS scenarios based on *Miscanthus* is low (≤1%), whereas those deploying irrigated poplar show a significant freshwater use (i.e., 40% of the SOS in BECCS-EXSITU-POP). We note that the deployment of NETs in water-stressed areas could have detrimental impacts at the regional level, even in the scenarios where freshwater consumption is low relative to the global PB.

BECCS and DACCS lead to low stratospheric ozone depletion (≤1% of the SOS) and land-system change (≤0.002% of the SOS). The land-system change impacts of BECCS are negligible—despite its high land-use requirements—because the LUC modeled in our scenarios does not involve the transformation of forested land, which is the only land type that the control variable of the land-system change PB considers^22^.

Finally, we found significant disparities between the human and planetary health rankings of NETs (Fig. 5b), the largest one corresponding to BECCS0-MISC (ranked 1 and 12 according to its human health and planetary impacts, respectively). LTSS-DACCS based on excess geothermal heat and DACCS powered by wind (LTSS and HTLS configurations) emerge as particularly appealing, averting substantial impacts on human health and the Earth system with minor detrimental side-effects.

## Discussion

Our analysis provides new insights into a much-debated question: are the risks and costs associated with the large-scale deployment of NETs worth their potential benefits? We found that DACCS and BECCS could—provided they overcome the current scale-up barriers—^41^ preserve a substantial number of years of healthy life that would otherwise be lost due to climate change. Notably, removing 5.9 net Gtonne/year CO_2_ could lead to net health improvements of a similar magnitude to the annual burden of common diseases. However, NETs would simultaneously generate some adverse health effects, mainly due to fine particulate matter formation. These unintended health impacts are negligible compared to the global effects of ambient air pollution, which currently reduce life expectancy by 3 years on average^42^. On the other hand, quantifying the NETs payback in terms of health externalities could incentivize their deployment, as the hidden monetized health benefits—often omitted in the economic analyses—are substantial (e.g., 142 US$/tonne CO_2_ captured for HTLS-DACCS powered with wind, which represents 81–117% of its estimated cost)^4^.

Focusing on the planetary health implications of the assessed NETs, only DACCS can avert the future climate change and ocean acidification impacts of past emissions without critically exacerbating the pressure on other Earth-system processes and concurrently preventing damage to the terrestrial biosphere integrity. In contrast, the biosphere integrity, nitrogen flow, and freshwater use would act as ecological bottlenecks for BECCS. Assessing the environmental impacts of NETs against regional PBs and the ecological limits to novel entities and aerosol loading—yet to be defined—^22^ could uncover other potential obstacles to their deployment. Nevertheless, DACCS will likely remain appealing given its positive effects on the two core planetary boundaries through which the others operate, i.e., climate change and biosphere integrity^22^. Moreover, future improvements in the energy efficiencies of DACCS systems could lower their health and environmental impacts; the long-term energy requirements of LTSS-DACCS could decrease by 38% (electricity) and 20% (heat) with respect to the values considered here^43^, whereas the energy demand of HTLS-DACCS could be reduced by optimizing the process parameters^4^. Furthermore, coupling HTLS-DACCS with electric furnaces could eliminate its dependence on natural gas.

NETs can prevent a temperature increase of 0.19 °C in our scenarios, but the combination of multiple climate change mitigation strategies might also allow us to meet the 1.5 °C target without resorting to NETs. It is challenging to evaluate whether the benefits of DACCS would outweigh its risks in such optimistic scenarios where emissions are significantly reduced. However, we are currently headed toward 2.7 °C, and the full implementation of pledges and binding targets would be conducive to 2.1 °C^44^. Therefore, unless efforts to reduce emissions are considerably intensified worldwide, NETs might become instrumental in meeting the Paris goals. While it is imperative to prioritize emissions reductions, the co-benefits and limited side-effects of DACCS might position this NET as an attractive option to counteract the historical responsibility of the most polluting countries and compensate for hard-to-abate emissions. Setting separate CDR and emissions reductions targets could prevent the potential mitigation deterrence effect of NETs^45^, which might discourage emissions reductions^46^ and allow a temporal temperature overshoot^47^.

From a broader perspective, our work provides a scientific basis to underpin current initiatives that complement the IPCC’s efforts by enlarging its scope beyond climate change. These include the ones led by the Earth Commission^48^, aiming to establish scientific guardrails for the Earth’s life-support systems, and the Global Commons Alliance^49^, focused on science-based action to protect people and the planet^50^. Furthermore, our results provide quantitative information about the implications of deploying NETs for several SDGs, besides SDG13 on climate action. Our human health analysis showcases how DACCS and BECCS could benefit SDG3 (good health and well-being) and SDG2 on zero hunger by reducing the risk of undernutrition and other health effects. Conversely, both NETs could hinder SDG6 (clean water and sanitation) and affect SDG15 (life on land) through their impacts on the freshwater use and biosphere integrity Earth-system processes, respectively. To assess the consequences of NETs for SDG7 (affordable and clean energy) and SDG14 (life below water), a broader set of indicators should be analyzed.

The performance of NETs will ultimately depend on their location; thus, a portfolio of negative emissions technologies and practices will probably be needed. Finding the optimal CDR roadmaps will require regional assessments and cooperation among countries to design sustainable supply chains for NETs, minimizing their collateral damage and costs by exploiting regional advantages. In this context, analyses like ours could bolster negotiations between international stakeholders, and guide climate change mitigation strategies aligned with sustainable development policies.

## Methods

### Life-cycle modeling

We applied the Life-Cycle Assessment (LCA) methodology^51,52^ to quantify the impacts of the studied NETs on human health and the Earth system throughout their entire life cycle. The functional unit—the net removal of 5.9 Gtonne/year CO_2_—corresponds to the average CDR rate in the SSP2-1.9 marker scenario during the period 2030–2100, excluding CDR in the agriculture, forestry and other land-use sector. Based on previous assessments^53–55^, we identified the demand for energy and land of DACCS and BECCS as the main factors constraining their deployment and verified that their global availability is sufficient to fulfill the functional unit (Supplementary Table 12). The impacts related to the functional unit were calculated by dividing the impacts of capturing 5.9 Gtonne/year CO_2_ by the CDR efficiency, ${{{{{{\rm{\eta }}}}}}}_{{{{{{\rm{C}}}}}}{{{{{{\rm{O}}}}}}}_{2}}$ (Supplementary Table 1), i.e., the ratio between the net amount of CO_2_ that is permanently sequestered—calculated as the total captured CO_2_ (${{{{{\rm{M}}}}}}{{{{{{\rm{C}}}}}}}_{{{{{{\rm{C}}}}}}{{{{{{\rm{O}}}}}}}_{2}}$) minus the overall CO_2_ emissions (${{{{{\rm{M}}}}}}{{{{{{\rm{E}}}}}}}_{{{{{{\rm{C}}}}}}{{{{{{\rm{O}}}}}}}_{2}}$),—and the amount captured$,{{{{{\rm{M}}}}}}{{{{{{\rm{C}}}}}}}_{{{{{{\rm{C}}}}}}{{{{{{\rm{O}}}}}}}_{2}}$ (Eq. 1).

1 $${\eta }_{C{O}_{2}}\,=\,\frac{{{MC}}_{C{O}_{2}}\,-\,M{E}_{C{O}_{2}}}{{{MC}}_{C{O}_{2}}}$$

We followed an attributional modeling approach where the background processes reflect the average market consumption mix^56^. Consequently, the results of our models change linearly with the net amount of CO_2_ removed. To address the multi-functionality of BECCS systems, which concurrently remove CO_2_ and generate electricity, we applied the system boundary expansion method. Thus, we consider that the produced electricity replaces electricity from the global mix projected for the SSP2-1.9 marker scenario in the period 2030–2100 (Supplementary Table 11), and the byproducts of the ex situ mineralization process substitute beneficiated iron ore and sand used as an inert filler. We also generated results for the BECCS scenarios disregarding the health and environmental credits (Supplementary Figs. 1, 2).

We assume that the impacts prevented by removing CO_2_ from the atmosphere are equal in magnitude to the impacts generated by emitting the same amount of CO_2_. However, several studies point out that global net negative emissions could weaken the natural carbon sinks^57–61^, reducing the NETs efficiency. Moreover, our LCA omits the impacts related to the infrastructure of NETs due to the lack of data. Nonetheless, previous studies suggest that the contribution of infrastructure to the total impacts of NETs might be minor. Notably, the impacts of constructing and decommissioning biomass power plants are negligible^62^, whereas the impacts related to the infrastructure of LTSS-DACCS are small relative to other life-cycle impacts^15^.

Our models were implemented in SimaPro 9.2^63^ and are based on generic—i.e., not geographically differentiated—data extracted from the Ecoinvent 3.5 database^64^ (cut-off by classification allocation method). Supplementary Tables 3–10 and Supplementary section 4 (Supplementary Tables 17, 18) provide further details on these LCA models and the assumptions made.

### Health impact assessment

Human health impacts are expressed in DALYs, which represent the years of healthy life lost due to either premature mortality or disability caused by prevalent disease or health conditions^65^. To estimate the health impacts in proportion to the global population, we considered the population prospects for SSP2^27,66^.

The non-climate health impacts were quantified following the Hierarchist cultural perspective of the ReCiPe 2016 endpoint method^67^, which integrates impacts over a 100-year time horizon, in accordance with the scientific consensus^68^. The environmental mechanisms leading to human health damage can be classified as global or regional depending on the scope of the health impacts. The health effects of climate change, stratospheric ozone depletion and ionizing radiation are global, whereas tropospheric ozone formation, water consumption and fine particulate matter formation cause regional health impacts. Stressors leading to human carcinogenic and non-carcinogenic toxicity impacts can affect human health at the global and regional levels, depending on their impact pathways. The metals compiled in Supplementary Table 2 contribute to at least 95% of the toxicity impacts across the studied scenarios. The USES-LCA 2.0 model^69^—on which the characterization factors of the toxicity stressors provided by ReCiPe are based—^67^ identifies human exposure via water consumption at the regional level as the main impact pathway for these metals within a 100-year time horizon. Therefore, we classified carcinogenic and non-carcinogenic toxicity as regional impacts.

The ReCiPe method does not provide spatially differentiated characterization factors for the stressors contributing to stratospheric ozone depletion and ionizing radiation^70^, which precludes the analysis of the geographical distribution of the associated global health impacts. To keep the analysis general, our models do not consider the NETs location; hence, we applied generic characterization factors.

Given the set S of stressors, we compute the non-climate health impacts of scenario *i* linked to environmental mechanism *e* (HI_i,e_) with Eq. 2, where EF_i,s_ represents the elementary flows (kg/year), and CF_e,s_, the characterization factors (DALY/kg). This equation applies to all the environmental mechanisms contributing to human health impacts except for climate change (cc).

2 $${HI}_{i,e}\,=\,\mathop{\sum} _{s\in {{{{{\rm{S}}}}}}}E{F}_{i,s}{{{{{\rm{\cdot }}}}}}C{F}_{e,s}\forall \,i,\,e\,\ne\, {cc}$$

Tang et al.^71^ derived spatially differentiated health damage factors from the models developed by the WHO^39^ for a subset of climate-related health risks—undernutrition, malaria, coastal floods, diarrhea, heat stress and dengue—under different adaptation measures. Other potential health impact pathways that are hard to predict, such as the effects of economic damage, or major heatwaves, are excluded from these models. Tang et al.^71^ estimated the aforementioned damage factors over the period 2000–2100; i.e., they do not refer to any specific year but to the whole period. These damage factors constitute an update of the factors used by the ReCiPe method to link global warming impacts to human health damage, which were derived from a previous WHO^72^ report by De Schryver et al.^73^ Thus, we calculated the impacts of climate change on human health with damage factors provided by Tang et al.^71^ for SSP2 (Supplementary Table 13).

We determined the climate-related health impacts of scenario *i* associated with health risk *h* in region *r* (${{{{{\rm{H}}}}}}{{{{{{\rm{I}}}}}}}_{{{{{{\rm{i}}}}}},{{{{{\rm{e}}}}}}\,=\,{{{{{\rm{cc}}}}}},{{{{{\rm{h}}}}}},{{{{{\rm{r}}}}}}}$) with Eq. 3 (list of countries and territories within the aggregated regions in Supplementary Table 14). Here, DF_h,r_ represents the damage factors proposed by Tang et al.^71^ (DALY/kg CO_2_-eq), and GWP_s_, the Global Warming Potential provided by the Hierarchist perspective of the ReCiPe method (kg CO_2_-eq/kg).

3 $$H{I}_{i,e,h,r}\,=\,D{F}_{h,r}{\,{\cdot }\,}\mathop{\sum} _{s\,\in\, {{{{{\rm{S}}}}}}}E{F}_{i,s}{\,{\cdot }\,}{GW}{P}_{s}\forall \,i,\,e\,=\,{cc},h,r$$

Neither the characterization factors used to quantify the climate and non-climate health impacts, nor the global burden of disease estimates of the WHO^65^ consider age-weighting or time discounting; therefore, they are comparable. Nevertheless, the health impacts related to the functional unit are aggregated over a 100-year time horizon—i.e., the impacts are associated with the net CO_2_ removed in 1 year, but they occur over a 100-year period. In contrast, the WHO's global burden of disease estimates refer to the health loss due to the prevalence of diseases and related premature deaths in one year (2019).

To quantify the health externalities of CDR, we applied the conversion factor proposed by Weidema^74,75^ (1 DALY = 74,000 €_2003_), which reflects the typical monetary value that society is willing to pay to preserve one DALY. Supplementary Section 3.2 provides further details about the monetization method.

### Earth-system impact assessment

We evaluated the impacts of our scenarios on the Earth-system processes identified by Steffen et al.^22^ as critical to preserving the Earth’s stability, excluding atmospheric aerosol loading and novel entities, for which global PBs are yet to be defined. We used the impact assessment method developed by Ryberg et al.^76^ complemented with the characterization factors proposed by Galán-Martín et al.^77^ to quantify the environmental impacts in terms of the control variables of the global PBs.

Ryberg et al.^76^ computed the climate change and ocean acidification characterization factors over a 300-year period. They made this choice because the net cumulative emissions of RCP2.6—the Representative Concentration Pathway taken as a basis to derive the characterization factors—between 2000 and 2300 lead to the stabilization of the atmospheric CO_2_ concentration at 361 ppm, a similar level to the climate change planetary boundary (350 ppm). Hence, the climate change impacts calculated for this time horizon reflect the tolerable level of impact that could prevent us from exceeding the climate change planetary boundary^76^.

We adjusted some of the characterization factors described by Ryberg et al.^76^ (Supplementary Table 15). The environmental impacts of scenario *i* on Earth-system process *j* (EI_i,j_) are calculated with Eq. (4), where CF_j,s_ is expressed in the units of the control variables of the PBs per unit of elementary flow EF_i,s_.

4 $$E{I}_{i,j}\,=\,\mathop{\sum} _{s\,\in\, {{{{{\rm{S}}}}}}}E{F}_{i,s}{\,{\cdot }\,}C{F}_{j,s}\forall \,i,\,j\,\ne\, {bi}$$

Ryberg et al.^76^ do not provide characterization factors for the biosphere integrity (*bi*). Thus, we estimated the impacts on the terrestrial biosphere with the characterization factors presented in^77^, which draw on the factors derived by Hanafiah et al.^78^ from mean species abundance statistics. As Eq. (5) shows, this method considers the two main drivers of terrestrial biodiversity loss^79,80^: greenhouse gas emissions (GHG) and land use (LU). Galán-Martín et al.^77^ estimated the characterization factors applied to the greenhouse gas elementary flows as the product of factor F_j,s_ obtained from^78^, and GWP_s_, estimated for a 100-year time horizon. Shorter time frames will result in lower avoided impacts on the terrestrial biosphere.

5 $$E{I}_{i,j}\,=\,\mathop{\sum}_{s\,\in\, {{{{{\rm{GHG}}}}}}}E{F}_{i,s}{\,{\cdot }\,}{GW}{P}_{s}{\,{\cdot }\,}{F}_{j,s}\,+\,\mathop{\sum}_{s\,\in\, {{{{{\rm{LU}}}}}}}E{F}_{i,s}{\,{\cdot }\,}C{F}_{j,s}\forall \,i,\,j\,=\,{bi}$$

Impacts are expressed as the percentage loss of mean species abundance, whereas the control variable proposed by Steffen et al.^22^ to measure functional diversity is the Biodiversity Intactness Index (BII)^81^. Given the lack of better estimates and the fact that biodiversity intactness can be expressed in terms of mean species abundance^82^, we assumed that the value of the biosphere integrity PB (10% decrease in BII) also applies to the loss of mean species abundance, in accordance with previous works^77,83,84^. Our analysis omits impacts on the freshwater and marine biosphere due to the absence of suitable impact assessment methods.

To assess the performance of our scenarios in terms of absolute sustainability, we calculated their impacts with respect to the size of the full SOS. According to Eq. (6), the impacts of scenario *i* on Earth-system process *j* relative to the size of the SOS (RI_i,j_) are computed as the ratio between their total impacts (EI_i,j_) and the SOS size, given by the absolute difference between the PB value (PB_j_) and the natural background level (NB_j_):

6 $$R{I}_{i,j}\left( \% \right)\,=\,\frac{E{I}_{i,j}}{\left|P{B}_{j}\,-\,N{B}_{j}\right|}{\,{\cdot }\,}100\,\forall \,i,\,j$$

Supplementary Table 16 provides the values of the PBs, the natural background level and the full SOS.

### Temperature in the baseline

We estimated the change in the global mean temperature in the baseline without NETs using the linear relationship between cumulative CO_2_ emissions and increase in global surface temperature considered by the IPCC^85^: 4.5 × 10^−4^ °C/Gtonne (best estimate, likely range: 2.7 × 10^−4^–6.3 × 10^−4^ °C/Gtonne). Accordingly, the rise in temperature with respect to pre-industrial levels in the baseline (which relies on the assumptions of climate change mitigation scenario SSP2-1.9/MESSAGE-GLOBIOM but does not include either DACCS or BECCS) is 1.52 °C by 2100, 0.19 °C [0.11–0.26 °C] higher than the temperature increase in the SSP2-1.9 marker scenario.

## Supplementary information

Supplementary Information

## Data Availability

The data supporting the findings of this study are available in the Supplementary Information document.

## References

[1] IPCC. *Climate Change 2022. Mitigation of Climate Change. Working Group III contribution to the Sixth Assessment Report of the Intergovernmental Panel on Climate Change*. (2022).

[2] Minx JC (2018). Negative emissions - Part 1: Research landscape and synthesis. Environ. Res. Lett..

[3] Fuss S (2018). Negative emissions - Part 2: Costs, potentials and side effects. Environ. Res. Lett..

[4] Keith DW, Holmes G, St. Angelo D, Heidel K (2018). A process for capturing CO_2_ from the atmosphere. Joule.

[5] Sagues WJ, Park S, Jameel H, Sanchez DL (2019). Enhanced carbon dioxide removal from coupled direct air capture-bioenergy systems. Sustain. Energy Fuels.

[6] Bhave A (2017). Screening and techno-economic assessment of biomass-based power generation with CCS technologies to meet 2050 CO_2_ targets. Appl. Energy.

[7] Hanna R, Abdulla A, Xu Y, Victor DG (2021). Emergency deployment of direct air capture as a response to the climate crisis. Nat. Commun..

[8] Realmonte G (2019). An inter-model assessment of the role of direct air capture in deep mitigation pathways. Nat. Commun..

[9] Marcucci A, Kypreos S, Panos E (2017). The road to achieving the long-term Paris targets: energy transition and the role of direct air capture. Clim. Change.

[10] Fuhrman J (2020). Food–energy–water implications of negative emissions technologies in a +1.5 °C future. Nat. Clim. Chang..

[11] Harper AB (2018). Land-use emissions play a critical role in land-based mitigation for Paris climate targets. Nat. Commun..

[12] Fajardy M, Mac Dowell N (2017). Can BECCS deliver sustainable and resource efficient negative emissions?. Energy Environ. Sci..

[13] Hanssen SV (2020). The climate change mitigation potential of bioenergy with carbon capture and storage. Nat. Clim. Chang..

[14] Galán-Martín Á (2021). Delaying carbon dioxide removal in the European Union puts climate targets at risk. Nat. Commun..

[15] Deutz S, Bardow A (2021). Life-cycle assessment of an industrial direct air capture process based on temperature–vacuum swing adsorption. Nat. Energy.

[16] Madhu K, Pauliuk S, Dhathri S, Creutzig F (2021). Understanding environmental trade-offs and resource demand of direct air capture technologies through comparative life-cycle assessment. Nat. Energy.

[17] Bello S, Galán-Martín Á, Feijoo G, Moreira MT, Guillén-Gosálbez G (2020). BECCS based on bioethanol from wood residues: Potential towards a carbon-negative transport and side-effects. Appl. Energy.

[18] Susmozas A, Iribarren D, Zapp P, Linβen J, Dufour J (2016). Life-cycle performance of hydrogen production via indirect biomass gasification with CO_2_ capture. Int. J. Hydrog. Energy.

[19] Terlouw T, Bauer C, Rosa L, Mazzotti M (2021). Life cycle assessment of carbon dioxide removal technologies: a critical review. Energy Environ. Sci..

[20] Heck V, Gerten D, Lucht W, Popp A (2018). Biomass-based negative emissions difficult to reconcile with planetary boundaries. Nat. Clim. Chang..

[21] Lade SJ (2020). Human impacts on planetary boundaries amplified by Earth system interactions. Nat. Sustain.

[22] Steffen W (2015). Planetary boundaries: Guiding human development on a changing planet. Science.

[23] Luderer G (2019). Environmental co-benefits and adverse side-effects of alternative power sector decarbonization strategies. Nat. Commun..

[24] Gibon T, Hertwich EG, Arvesen A, Singh B, Verones F (2017). Health benefits, ecological threats of low-carbon electricity. Environ. Res. Lett..

[25] Creutzig F (2019). The mutual dependence of negative emission technologies and energy systems. Energy Environ. Sci..

[26] Rogelj J (2018). Scenarios towards limiting global mean temperature increase below 1.5 °C. Nat. Clim. Chang..

[27] International Institute for Applied Systems Analysis. SSP Database. https://tntcat.iiasa.ac.at/SspDb/dsd?Action=htmlpage&page=40 last accessed: 15.11.21.

[28] Riahi K (2017). The shared socioeconomic pathways and their energy, land use, and greenhouse gas emissions implications: an overview. Glob. Environ. Chang.

[29] Fricko O (2017). The marker quantification of the shared socioeconomic pathway 2: a middle-of-the-road scenario for the 21st century. Glob. Environ. Chang.

[30] Bauer, C. et al. Energy from the Earth. Deep geothermal as a resource for the future? 10.3929/ethz-a-010277690 (2015).

[31] IEA Greenhouse Gas R&D Programme. Biomass CCS Study. https://www.globalccsinstitute.com/archive/hub/publications/98606/biomass-ccs-study.pdf (2009).

[32] IEA. The Future of Hydrogen. Seizing today’s opportunities. https://read.oecd-ilibrary.org/energy/thefuture-of-hydrogen_1e0514c4-en#page2 (2019).

[33] Qin Z, Dunn JB, Kwon H, Mueller S, Wander MM (2016). Soil carbon sequestration and land use change associated with biofuel production: empirical evidence. GCB Bioenergy.

[34] Snæbjörnsdóttir SÓ (2020). Carbon dioxide storage through mineral carbonation. Nat. Rev. Earth Environ..

[35] Romão I, Nduagu E, Fagerlund J, Gando-Ferreira LM, Zevenhoven R (2012). CO_2_ fixation using magnesium silicate minerals. Part 2: energy efficiency and integration with iron-and steelmaking. Energy.

[36] Fagerlund J, Nduagu E, Romão I, Zevenhoven R (2012). CO_2_ fixation using magnesium silicate minerals part 1: Process description and performance. Energy.

[37] Arpagaus C, Bless F, Uhlmann M, Schiffmann J, Bertsch SS (2018). High temperature heat pumps: Market overview, state of the art, research status, refrigerants, and application potentials. Energy.

[38] World Health Organization. *Global Health Estimates 2019: Disease burden by Cause, Age, Sex, by Country and by Region, 2000–2019*. https://www.who.int/data/gho/data/themes/mortality-and-global-health-estimates/global-health-estimates-leading-causes-of-dalys (2020).

[39] World Health Organization. Quantitative risk assessment of the effects of climate change on selected causes of death, 2030s and 2050s. https://apps.who.int/iris/bitstream/handle/10665/134014/9789241507691_eng.pdf?sequence=1&isAllowed=y (2014).

[40] Partnerships in Environmental Management for the Seas of East Asia. *Sustain. Dev. Strat. Seas of East Asia*. (2015).

[41] Nemet GF (2018). Negative emissions - Part 3: Innovation and upscaling. Environ. Res. Lett..

[42] Lelieveld J (2020). Loss of life expectancy from air pollution compared to other risk factors: a worldwide perspective. Cardiovasc. Res..

[43] Beuttler C, Charles L, Wurzbacher J (2019). The Role of Direct Air Capture in Mitigation of Anthropogenic Greenhouse Gas Emissions. Front. Clim..

[44] Climate Action Tracker. Warming projections global update. https://climateactiontracker.org/documents/997/CAT_2021-11-09_Briefing_Global-Update_Glasgow2030CredibilityGap.pdf (2021).

[45] Grant N, Hawkes A, Mittal S, Gambhir A (2021). Confronting mitigation deterrence in lowcarbon scenarios. Environ. Res. Lett..

[46] Pradhan S (2021). Effects of direct air capture technology availability on stranded assets and committed emissions in the power sector. Front. Clim..

[47] Rogelj J (2019). A new scenario logic for the Paris Agreement long-term temperature goal. Nature.

[48] Earth Commission. https://earthcommission.org/ (last accessed: 16.07.21).

[49] Global Commons Alliance. https://globalcommonsalliance.org/ (last accessed: 16.07.21).

[50] Rockström J (2021). Identifying a safe and just corridor for people and the planet. Earth’s Futur.

[51] ISO. ISO 14040. Environmental Management — Life Cycle Assessment — Principles and Framework. https://www.iso.org/standard/37456.html (2006).

[52] ISO. ISO 14044. Environmental management — Life cycle assessment — Requirements and guidelines. https://www.iso.org/standard/38498.html (2006).

[53] Smith P (2016). Biophysical and economic limits to negative CO_2_ emissions. Nat. Clim. Chang..

[54] Chatterjee S, Huang KW (2020). Unrealistic energy and materials requirement for direct air capture in deep mitigation pathways. Nat. Commun..

[55] Realmonte G (2020). Reply to “High energy and materials requirement for direct air capture calls for further analysis and R&D”. Nat. Commun..

[56] European Commission - Joint Research Centre - Institute for Environment and Sustainability. International Reference Life Cycle Data System (ILCD) Handbook - General guide for Life Cycle Assessment - Detailed guidance. https://eplca.jrc.ec.europa.eu/uploads/ILCD-Handbook-General-guide-for-LCADETAILED-GUIDANCE-12March2010-ISBN-fin-v1.0-EN.pdf (2010).

[57] Zickfeld K, MacDougall AH, Damon Matthews H (2016). On the proportionality between global temperature change and cumulative CO_2_ emissions during periods of net negative CO_2_ emissions. Environ. Res. Lett..

[58] Jones CD (2016). Simulating the Earth system response to negative emissions. Environ. Res. Lett..

[59] Vichi M, Navarra A, Fogli PG (2013). Adjustment of the natural ocean carbon cycle to negative emission rates. Clim. Change.

[60] Keller DP (2018). The effects of carbon dioxide removal on the carbon cycle. Curr. Clim. Chang. Rep..

[61] Zickfeld K, Azevedo D, Mathesius S, Matthews HD (2021). Asymmetry in the climate–carbon cycle response to positive and negative CO_2_ emissions. Nat. Clim. Chang..

[62] Bauer, C. Life Cycle Assessment of Fossil and Biomass Power Generation Chains. https://www.osti.gov/etdeweb/servlets/purl/21369007 (2008).

[63] SimaPro. https://simapro.com/ (last accessed: 12/02/2021).

[64] Wernet G (2016). The ecoinvent database version 3 (part I): overview and methodology. Int. J. Life Cycle Assess..

[65] World Health Organization. WHO methods and data sources for global burden of disease estimates 2000-2019. https://cdn.who.int/media/docs/default-source/gho-documents/global-health-estimates/ghe2019_daly-methods.pdf?sfvrsn=31b25009_7 (2020).

[66] KC S, Lutz W (2017). The human core of the shared socioeconomic pathways: Population scenarios by age, sex and level of education for all countries to 2100. Glob. Environ. Chang.

[67] Huijbregts, M.A.J., et al. *ReCiPe 2016 v1.1. A harmonized life cycle impact assessment method at midpoint and endpoint level. Report I: Characterization*. (2017).

[68] Verones F (2020). LC-IMPACT: a regionalized life cycle damage assessment method. J. Ind. Ecol..

[69] Van Zelm R, Huijbregts MAJ, Van De Meent D (2009). USES-LCA 2.0—a global nested multi-media fate, exposure, and effects model. Int. J. Life Cycle Assess..

[70] Huijbregts MAJ (2017). ReCiPe2016: a harmonised life cycle impact assessment method at midpoint and endpoint level. Int. J. Life Cycle Assess..

[71] Tang L, Furushima Y, Honda Y, Hasegawa T, Itsubo N (2019). Estimating human health damage factors related to CO_2_ emissions by considering updated climate-related relative risks. Int. J. Life Cycle Assess..

[72] World Health Organization. Comparative quantification of health risks. Global and regional burden of disease attributable to selected major risk factors. https://apps.who.int/iris/handle/10665/42770 (2004).

[73] De Schryver AM, Brakkee KW, Goedkoop MJ, Huijbregts MAJ (2009). Characterization factors for global warming in life cycle assessment based on damages to humans and ecosystems. Environ. Sci. Technol..

[74] Weidema BP (2015). Comparing three life cycle impact assessment methods from an endpoint perspective. J. Ind. Ecol..

[75] Weidema BP (2009). Using the budget constraint to monetarise impact assessment results. Ecol. Econ..

[76] Ryberg MW, Owsianiak M, Richardson K, Hauschild MZ (2018). Development of a life-cycle impact assessment methodology linked to the Planetary Boundaries framework. Ecol. Indic..

[77] Galán-Martín Á (2021). Sustainability footprints of a renewable carbon transition for the petrochemical sector within planetary boundaries. One Earth.

[78] Hanafiah MM, Hendriks AJ, Huijbregts MAJ (2012). Comparing the ecological footprint with the biodiversity footprint of products. J. Clean. Prod..

[79] Newbold T (2016). Has land use pushed terrestrial biodiversity beyond the planetary boundary? A global assessment. Science.

[80] Barnosky AD (2012). Approaching a state shift in Earth’s biosphere. Nature.

[81] Scholes RJ, Biggs R (2005). A biodiversity intactness index. Nature.

[82] Schipper AM (2020). Projecting terrestrial biodiversity intactness with GLOBIO 4. Glob. Chang. Biol..

[83] D’Angelo SC (2021). Planetary boundaries analysis of low-carbon ammonia production routes. ACS Sustain. Chem. Eng..

[84] Lucas E, Guo M, Guillén-Gosálbez G (2021). Optimising diets to reach absolute planetary environmental sustainability through consumers. Sustain. Prod. Consum.

[85] IPCC. *Climate Change 2021. The Physical Science Basis. Working Group I contribution to the Sixth Assessment Report of the Intergovernmental Panel on Climate Change*. (2021).

