# Supplementary material for: Human and planetary health implications of negative emissions technologies
Source: Nat Commun. 2022 May 9;13:2535. doi: 10.1038/s41467-022-30136-7 (PMC9085842; doi:10.1038/s41467-022-30136-7)
Supplement: Supplementary file 1 — Supplementary Information [file 41467_2022_30136_MOESM1_ESM.pdf]

Human and planetary health implications of negative emissions technologies

Supplementary Information

Selene Cobo[1], Ángel Galán-Martín[2,3], Victor Tulus[1], Mark A.J. Huijbregts[4] and Gonzalo Guillén-Gosálbez[1]*

[1]Department of Chemistry and Applied Biosciences, Institute for Chemical and Bioengineering, ETH Zürich, Zürich, Switzerland.

[2]Department of Chemical, Environmental and Materials Engineering, Universidad de Jaén, Jaén, Spain.

[3]Center for Advanced Studies in Earth Sciences, Energy and Environment, Universidad de Jaén, Jaén, Spain.

[4]Department of Environmental Science, Radboud Institute for Biological and Environmental Sciences, Radboud University, Nijmegen, the Netherlands.

*gonzalo.guillen.gosalbez@chem.ethz.ch

This document describes the models and data sources, further clarifies the methods, and provides additional results.

Table of Contents

1. Supplementary results 3
   1.1 Impact assessment 3
      1.1.1 Health and environmental impacts without credits 3
      1.1.2 Health and environmental impacts of the in situ sequestration processes 3
      1.1.3 Breakdown of health and environmental impacts 3
      1.1.4 Externalities 10
   1.2 CDR efficiency 13
   1.3 Toxicity stressors 14
2. Life cycle models 15
   2.1 Direct Air Capture 15
   2.2 Bioenergy with Carbon Capture 16
   2.3 Transport and sequestration 19
   2.4 Electricity mix 21
   2.5 Potential deployment constraints 22
3. Supplementary methods 23
   3.1 Human health impact assessment 23
      3.1.1 Health damage factors of CO_2 emissions 23
   3.2 Estimation of externalities 25
   3.3 Earth-system impact assessment 26
4. Assumptions and limitations 29
   4.1 Life cycle models 29
   4.2 Impact assessment method 31
5. Supplementary references 32

1. Supplementary results

The results we present here help better understand the findings described in the main manuscript.

1.1 Impact assessment

1.1.1 Health and environmental impacts without credits

We generated additional results without considering the health and environmental credits. Accordingly, the health and environmental impacts depicted in Supplementary Figures 1 and 2 exclude the impacts prevented by substituting the electricity from the global mix with the electricity generated in the BECCS scenarios, as well as the impacts prevented by replacing beneficiated iron ore and sand with the byproducts of the ex situ mineralization.

Comparing these results to the impacts shown in Figures 3 and 5 of the main manuscript (which account for the avoided impacts), we can see that the assumption that the generated electricity replaces electricity from the global mix has a substantial influence on the results; the scenarios are ranked differently according to the human health impacts if the electricity credits are omitted.

1.1.2 Health and environmental impacts of the in situ sequestration processes

Supplementary Figures 3 and 4 display the health and environmental impacts of the scenarios based on the studied *in situ* sequestration options (geological sequestration at high pressure and mineralization based on freshwater and seawater).

The scenarios relying on mineralization with seawater lead to the lowest health impacts because of their low electricity and freshwater requirements. Mineralization with freshwater is the most damaging sequestration option to human health in all the studied scenarios owing to the health impacts related to its high freshwater use.

The scenarios relying on geological sequestration at high pressure generate more impact across the studied Earth-system processes – excluding global freshwater use, for which mineralization with freshwater is the most detrimental sequestration option – because of its higher electricity requirements. The impact of the in situ mineralization processes is quite similar across all the Earth-system processes – excluding freshwater use –, irrespective of the water source.

1.1.3 Breakdown of health and environmental impacts

The health and environmental impacts attributed to the unit processes that integrate the NETs systems, and their inputs and outputs, are depicted in Supplementary Figures 5 and 6. The results of the HTLS-DACCS, LTSS-DACCS, BECCS0 and BEDACCS scenarios represent the average impacts estimated for the NETs deploying the three in situ sequestration options. Overall, the required energy and biomass are the principal contributors to the detrimental health and environmental impacts of the DACCS and BECCS scenarios, respectively.

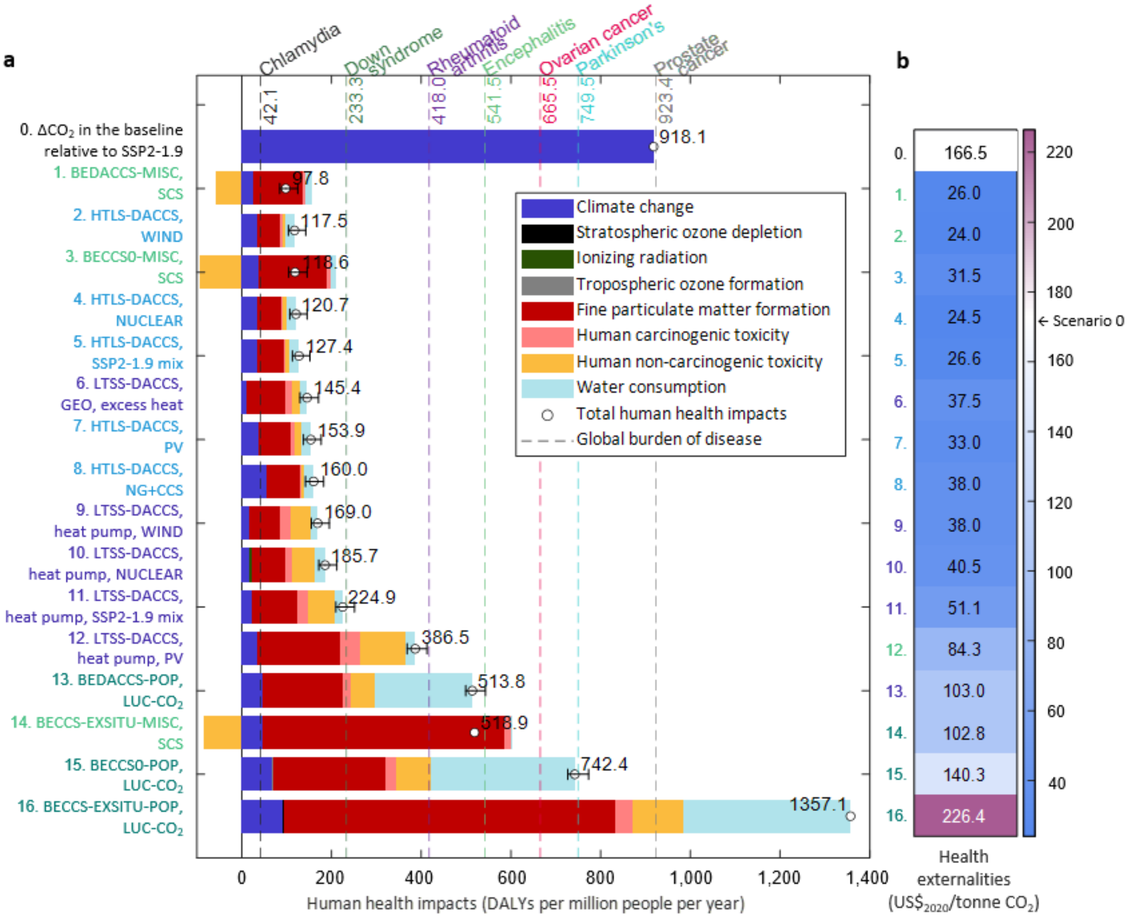

Supplementary Fig. 1. Health impacts, excluding the health credits of the produced electricity and the byproducts of the ex situ mineralization in the BECCS scenarios. a Contribution of environmental mechanisms to the total health impacts, expressed in Disability-Adjusted life years (DALYs) per million people per year. Scenarios 1-16 comprise High-Temperature Liquid Sorbent (HTLS) and Low-Temperature Solid Sorbent (LTSS) DACCS – powered by natural gas with carbon capture and storage (NG+CCS), wind, solar photovoltaic (PV), nuclear, geothermal (GEO), or the global electricity mix deployed in the SSP2-1.9 marker scenario without NETs –, the basic BECCS scenarios (BECCS0) deploying *Miscanthus* (MISC) or poplar (POP) – assuming either Soil Carbon Sequestration (SCS) or land-use change (LUC) –, the hybrid BEDACCS configurations integrating BECCS0 and LTSS-DACCS, and the BECCS scenarios where CO_2 is mineralized ex situ (BECCS-EXSITU). Scenarios 1-16 are ranked by the total health impacts, scenario 1 is the best. We show the global burden of certain diseases in 2019[1] for reference. The black bars indicate the health impact range of the scenarios based on the in situ sequestration options, i.e., geological sequestration at high pressure and mineral carbonation with freshwater (upper bound) or seawater (lower bound). **b** Health externalities, expressed in US$$_{2020}$ per gross tonne CO_2 captured (scenarios 1-16) or emitted (scenario 0).

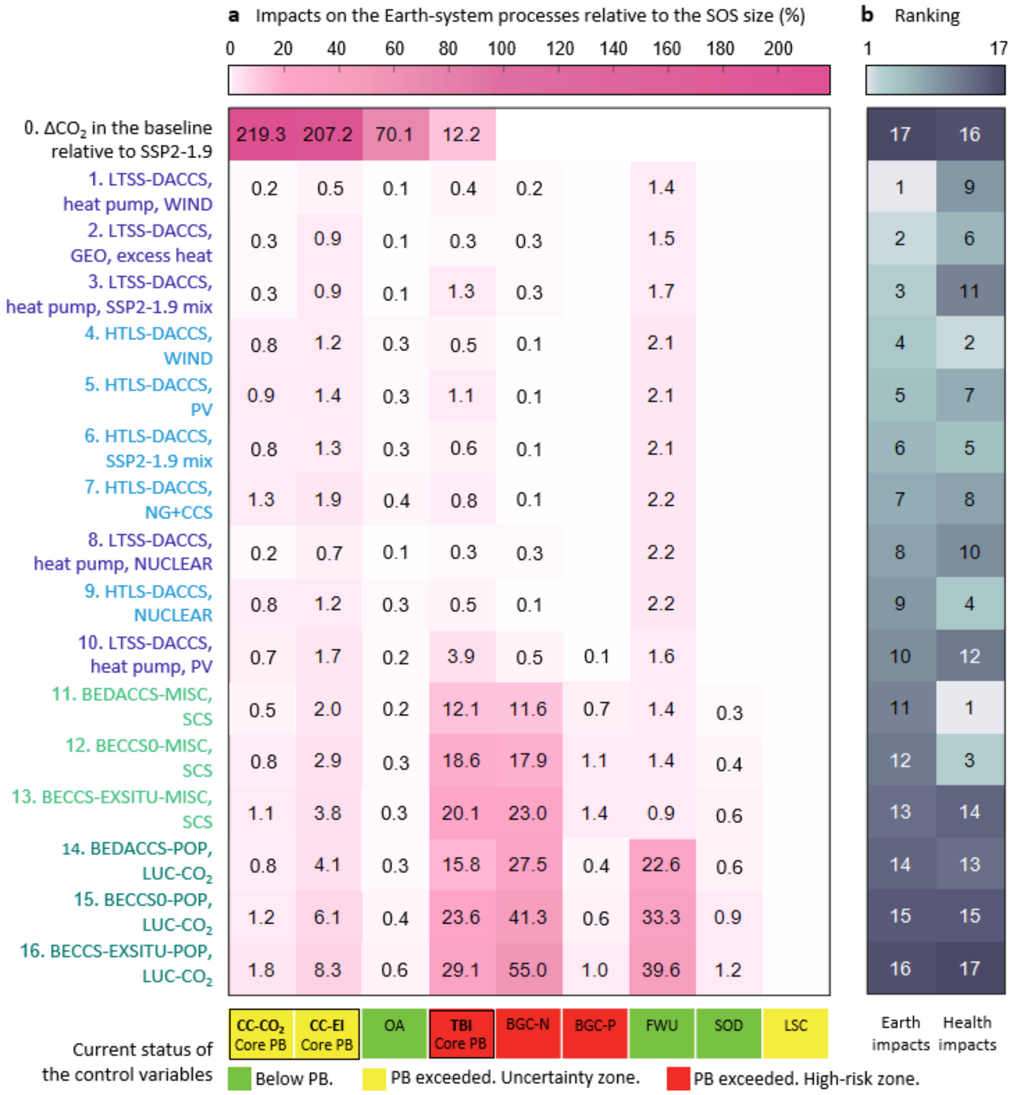

Supplementary Fig. 2. Impacts on the Earth-system processes – excluding the environmental credits of the produced electricity and the byproducts of the ex situ mineralization in the BECCS scenarios – and ranking of scenarios by impacts on human health and the Earth system. a Impacts on Earth-system processes expressed as a percentage of the size of the Safe Operating Space (SOS). The impacts on the following Earth-system processes were assessed: climate change – considering atmospheric CO_2 concentration (CC-CO_2) and energy imbalance (CC-EI) as control variables –, ocean acidification (OA), terrestrial biosphere integrity (TBI), global biogeochemical flows – considering the application rate of intentionally fixed reactive nitrogen to the agricultural system (BGC-N) and phosphorus flows from freshwater into the ocean (BGC-P) as control variables –, global freshwater use (FWU), stratospheric ozone depletion (SOD), and global land-system change (LSC). Scenarios 1-16 comprise High-Temperature Liquid Sorbent (HTLS) and Low-Temperature Solid Sorbent (LTSS) DACCS – powered by natural gas with carbon capture and storage (NG+CCS), wind, solar photovoltaic (PV), nuclear, geothermal (GEO), or the global electricity mix deployed in the SSP2-1.9 marker scenario without NETs –, the basic BECCS scenarios (BECCS0) deploying *Miscanthus* (MISC) or poplar (POP) – assuming either Soil Carbon Sequestration (SCS) or land-use change (LUC) –, the hybrid BEDACCS configurations integrating BECCS0 and LTSS-DACCS, and the BECCS scenarios where CO_2 is mineralized ex situ (BECCS-EXSITU). The values of empty cells range between 0 and 0.05%. We show the current level of the control variables for the Planetary Boundaries (PBs) of the studied Earth-system processes below using a qualitative color code, according to the PB framework.[2] **b** Ranking of scenarios by health impacts and maximum impacts across Earth-system processes relative to the SOS size, scenario 1 is the best.

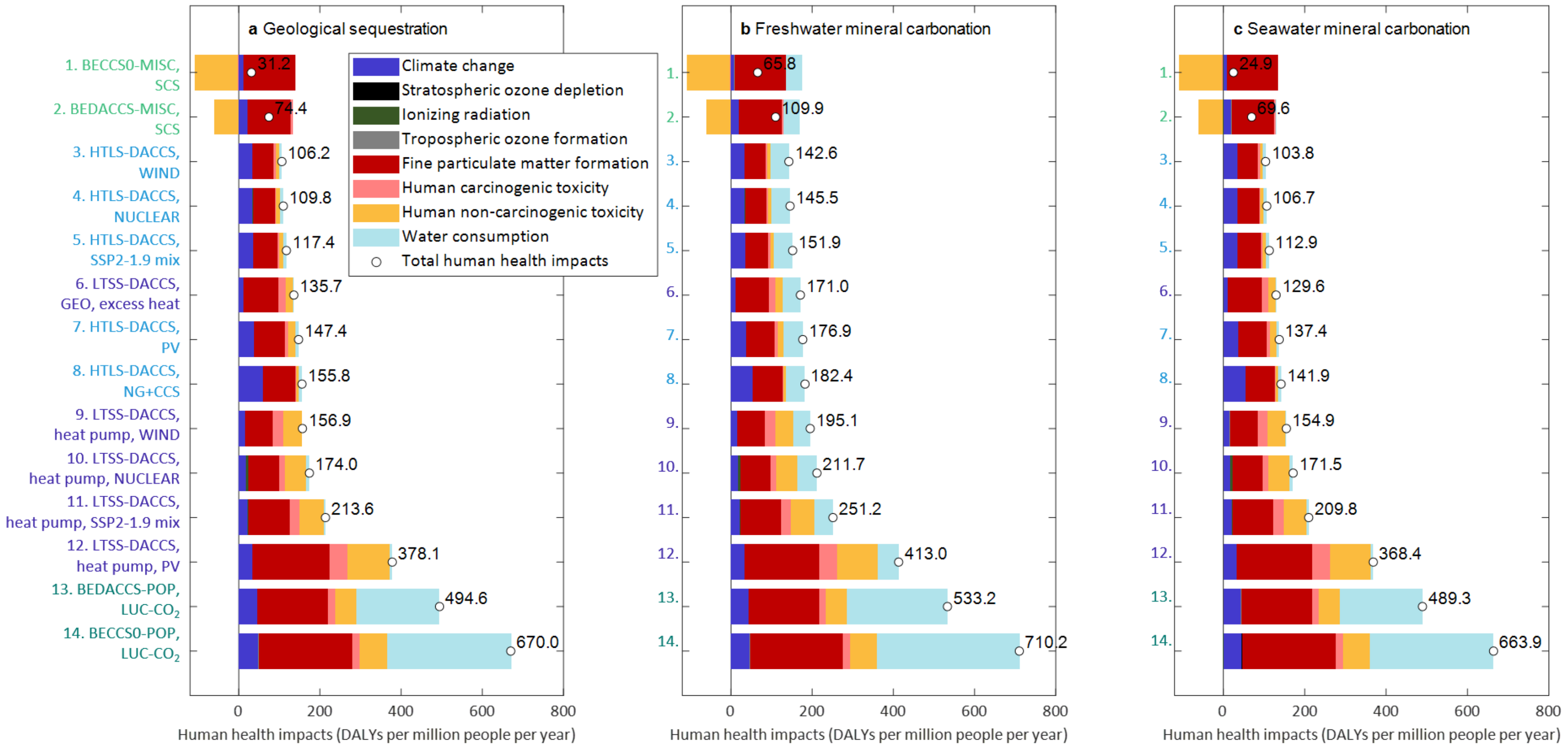

Supplementary Fig. 3. Health impacts (disaggregated by environmental mechanisms) of the scenarios based on the in situ sequestration alternatives: a Geological sequestration at high pressure. **b** Freshwater mineral carbonation. **c** Seawater mineral carbonation. The scenarios are ranked by the average health impacts, expressed in Disability-Adjusted life years (DALYs) per million people per year. Scenarios 1-16 comprise High-Temperature Liquid Sorbent (HTLS) and Low-Temperature Solid Sorbent (LTSS) DACCS – powered by natural gas with carbon capture and storage (NG+CCS), wind, solar photovoltaic (PV), nuclear, geothermal (GEO), or the global electricity mix deployed in the SSP2-1.9 marker scenario without NETs –, the basic BECCS scenarios (BECCS0) deploying *Miscanthus* (MISC) or poplar (POP) – assuming either Soil Carbon Sequestration (SCS) or land-use change (LUC) –, the hybrid BEDACCS configurations integrating BECCS0 and LTSS-DACCS, and the BECCS scenarios where CO_2 is mineralized ex situ (BECCS-EXSITU). Scenario 1 is the best.

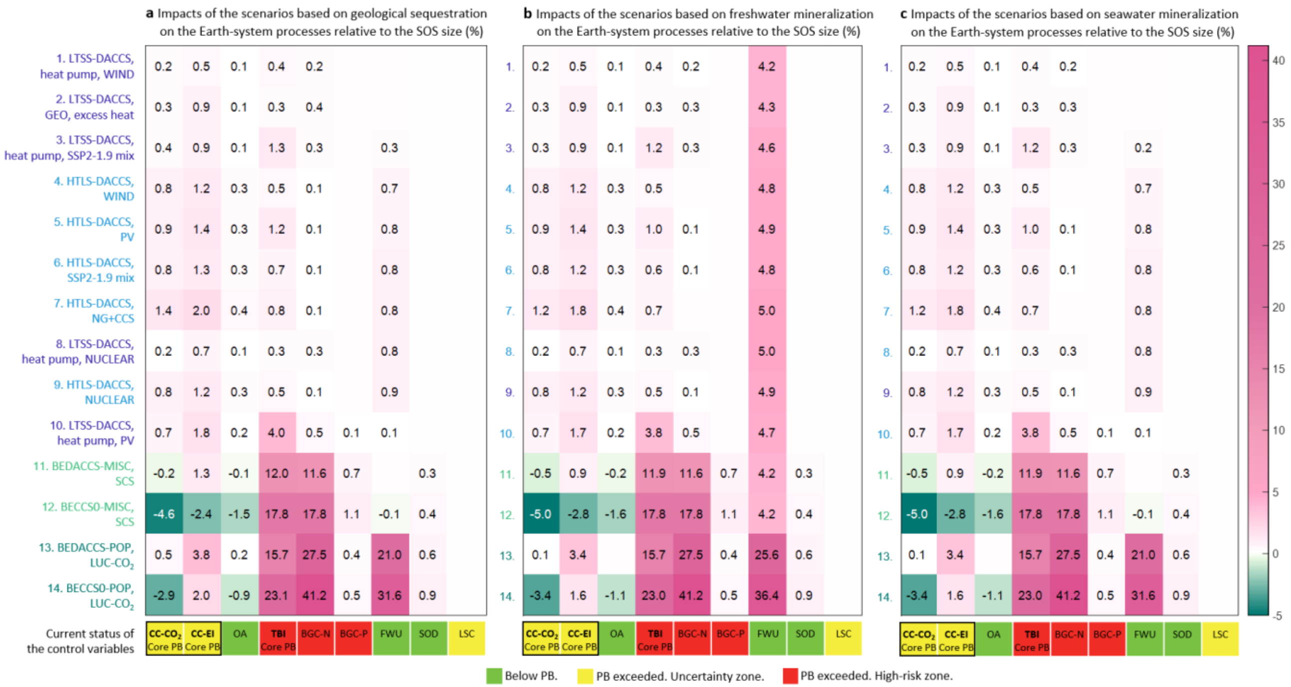

Supplementary Fig. 4. Impacts on the Earth-system processes – expressed as a percentage of the size of the Safe Operating Space (SOS) – of the scenarios based on the in situ sequestration alternatives: a Geological sequestration at high pressure. **b** Freshwater mineral carbonation. **c** Seawater mineral carbonation. The impacts on the following Earth-system processes were assessed: climate change – considering atmospheric CO_2 concentration (CC-CO_2) and energy imbalance (CC-EI) as control variables –, ocean acidification (OA), terrestrial biosphere integrity (TBI), global biogeochemical flows – considering the application rate of intentionally fixed reactive nitrogen to the agricultural system (BGC-N) and phosphorus flows from freshwater into the ocean (BGC-P) as control variables –, global freshwater use (FWU), stratospheric ozone depletion (SOD), and global land-system change (LSC). Scenarios 1-16 comprise High-Temperature Liquid Sorbent (HTLS) and Low-Temperature Solid Sorbent (LTSS) DACCS – powered by natural gas with carbon capture and storage (NG+CCS), wind, solar photovoltaic (PV), nuclear, geothermal (GEO), or the global electricity mix deployed in the SSP2-1.9 marker scenario without NETs –, the basic BECCS scenarios (BECCS0) deploying *Miscanthus* (MISC) or poplar (POP) – assuming either Soil Carbon Sequestration (SCS) or land-use change (LUC) –, the hybrid BEDACCS configurations integrating BECCS0 and LTSS-DACCS, and the BECCS scenarios where CO_2 is mineralized ex situ (BECCS-EXSITU). We show the current level of the control variables for the Planetary Boundaries (PBs) of the studied Earth-system processes below using a color code, according to the PB framework.[2] The scenarios are ranked by the maximum impacts across Earth-system processes (average of sequestration alternatives), scenario 1 is the best. The values of empty cells range between $1{\cdot}10^{-4}$ and 0.08%.

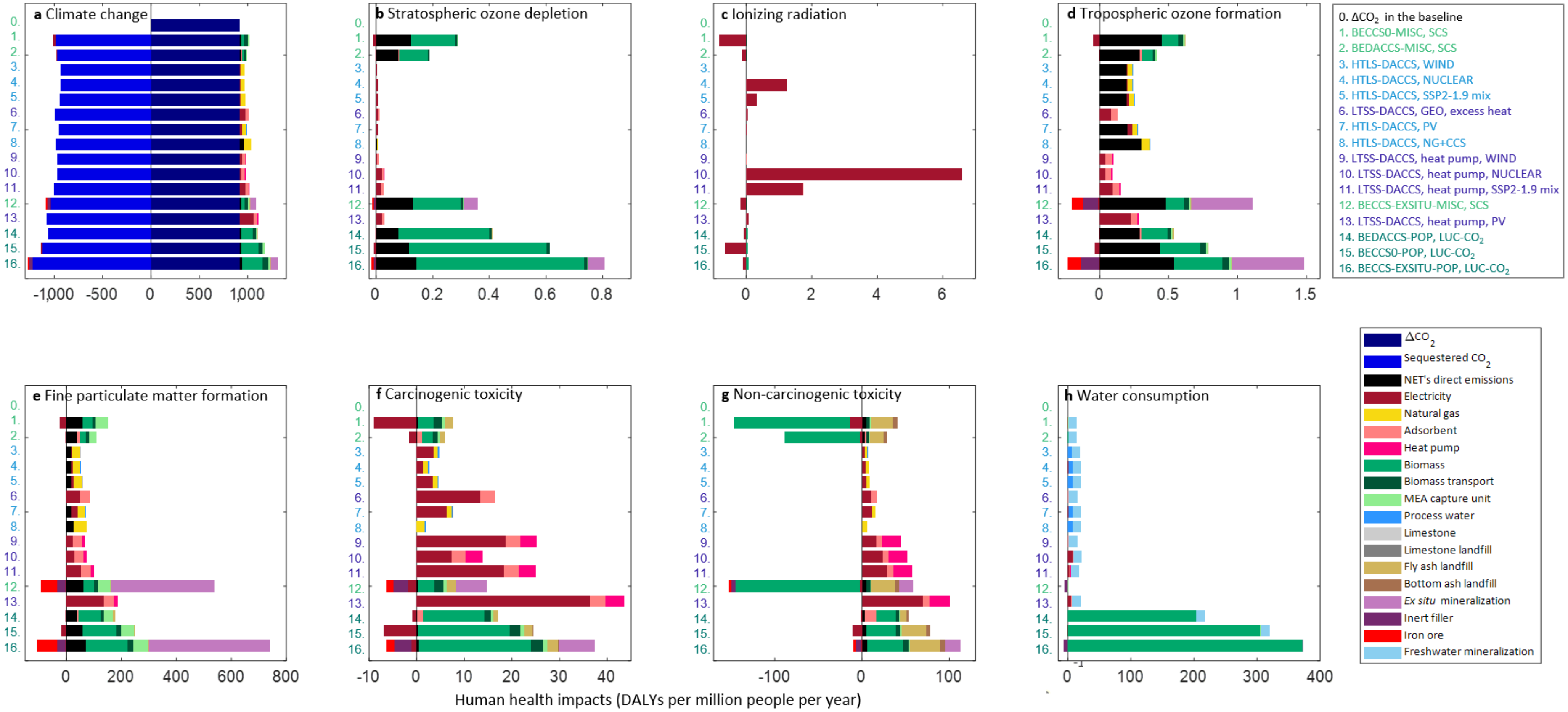

Supplementary Fig. 5. Breakdown of human health impacts due to a Climate change. **b** Stratospheric ozone depletion. **c** Ionizing radiation. **d** Tropospheric ozone formation. **e** Fine particulate matter formation. **f** Carcinogenic toxicity. **g** Non-carcinogenic toxicity. **h** Water consumption. Scenarios 1-16 comprise High-Temperature Liquid Sorbent (HTLS) and Low-Temperature Solid Sorbent (LTSS) DACCS – powered by natural gas with carbon capture and storage (NG+CCS), wind, solar photovoltaic (PV), nuclear, geothermal (GEO), or the global electricity mix deployed in the SSP2-1.9 marker scenario without NETs –, the basic BECCS scenarios (BECCS0) deploying *Miscanthus* (MISC) or poplar (POP) – assuming either Soil Carbon Sequestration (SCS) or land-use change (LUC) –, the hybrid BEDACCS configurations integrating BECCS0 and LTSS-DACCS, and the BECCS scenarios where CO_2 is mineralized ex situ (BECCS-EXSITU). Scenarios 1-16 are ranked by the total health impacts, expressed in expressed in Disability-Adjusted life years (DALYs) per million people per year. Scenario 1 is the best.

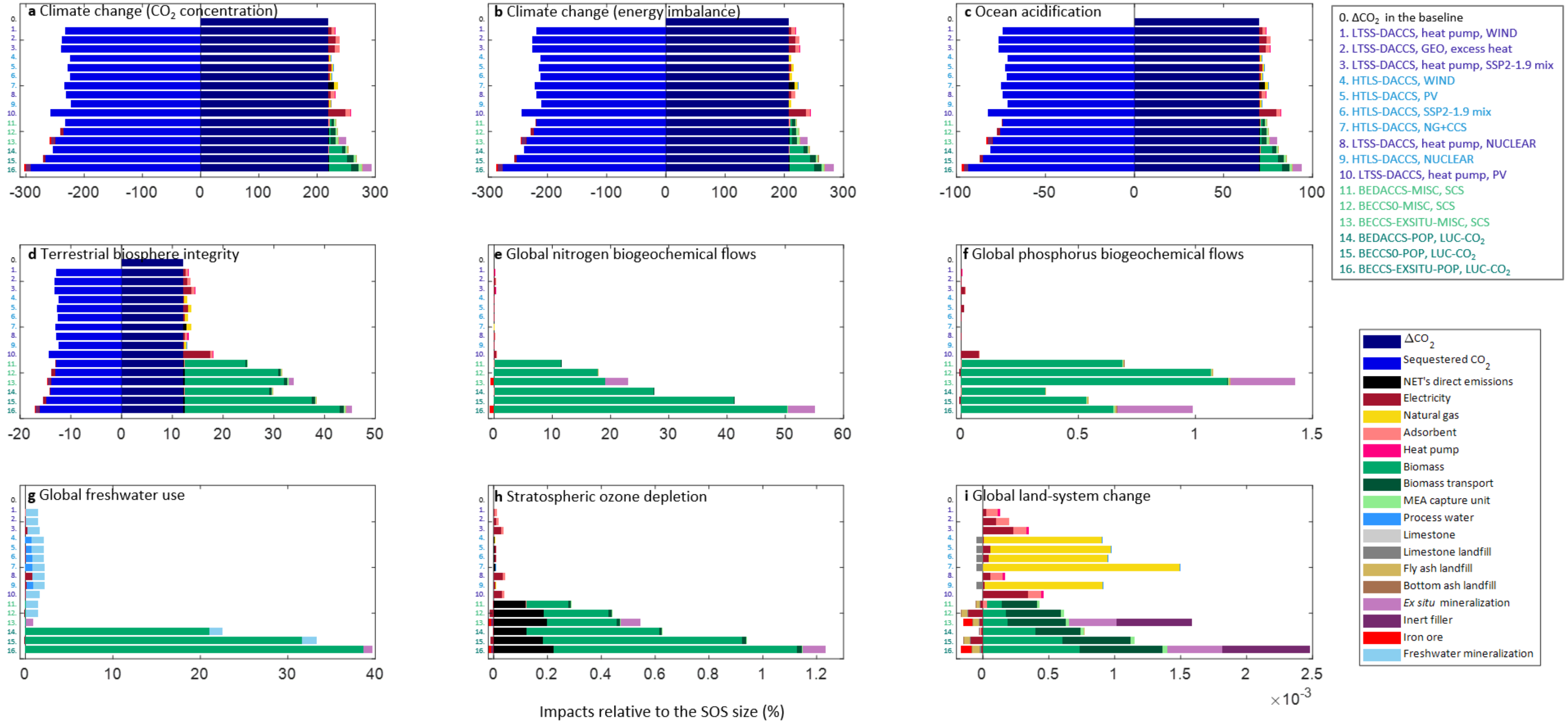

Supplementary Fig. 6. Breakdown of impacts on these Earth-system processes: a Climate change (control variable: CO_2 concentration). **b** Climate change (control variable: energy imbalance). **c** Ocean acidification. **d** Terrestrial biosphere integrity. **e** Global biogeochemical flows (control variable: application rate of intentionally fixed reactive nitrogen to the agricultural system). **f** Global biogeochemical flows (control variable: phosphorus flows from freshwater into the ocean). **g** Global freshwater use. **h** Stratospheric ozone depletion. **i** Global land-system change. Scenarios 1-16 are ranked by the maximum impacts across Earth-system processes, expressed as a percentage of the size of the Safe Operating Space (SOS). Scenarios 1-16 comprise High-Temperature Liquid Sorbent (HTLS) and Low-Temperature Solid Sorbent (LTSS) DACCS – powered by natural gas with carbon capture and storage (NG+CCS), wind, solar photovoltaic (PV), nuclear, geothermal (GEO), or the global electricity mix deployed in the SSP2-1.9 marker scenario without NETs –, the basic BECCS scenarios (BECCS0) deploying *Miscanthus* (MISC) or poplar (POP) – assuming either Soil Carbon Sequestration (SCS) or land-use change (LUC) –, the hybrid BEDACCS configurations integrating BECCS0 and LTSS-DACCS, and the BECCS scenarios where CO_2 is mineralized ex situ (BECCS-EXSITU). Scenario 1 is the best.

1.1.4 Externalities

Supplementary Figure 7 provides the health externalities of the NETs configurations based on the in situ sequestration options (geological sequestration at high pressure and in situ mineralization with freshwater or seawater).

Note that in the main manuscript, we only reported the externalities linked to human health. Here we expand the analysis to cover the indirect costs associated with the other areas of protection, namely ecosystems quality and resource availability, to investigate whether they could offset the prevented health externalities. The externalities associated with the potential economic losses caused by climate change (e.g., infrastructure damaged due to extreme weather events, or the implementation of adaptation measures) are omitted from this assessment.

Supplementary Figure 8 shows the total monetized impacts of the studied NETs. Overall, the externalities associated with CO_2 emissions can be reduced relative to the baseline with all the NETs except for the BECCS0 and BECCS-EXSITU configurations based on poplar, given their substantial impact on human health and ecosystems. While CDR prevents the harmful effects of CO_2 on ecosystems, the extensive land use of BECCS leads to ecosystem damage and hence, additional externalities. On the contrary, the DACCS configurations reduce the impacts on ecosystems with respect to the baseline and thus, the associated externalities are negative.

The difference between the human health externalities and the total externalities varies across scenarios. This mismatch is minor in the LTSS-DACCS scenarios. However, more significant mismatches are observed for HTLS-DACCS – because of the large impact on resource availability associated with its natural gas consumption – and the BECCS configurations, due to the impacts of biomass cultivation on ecosystems.

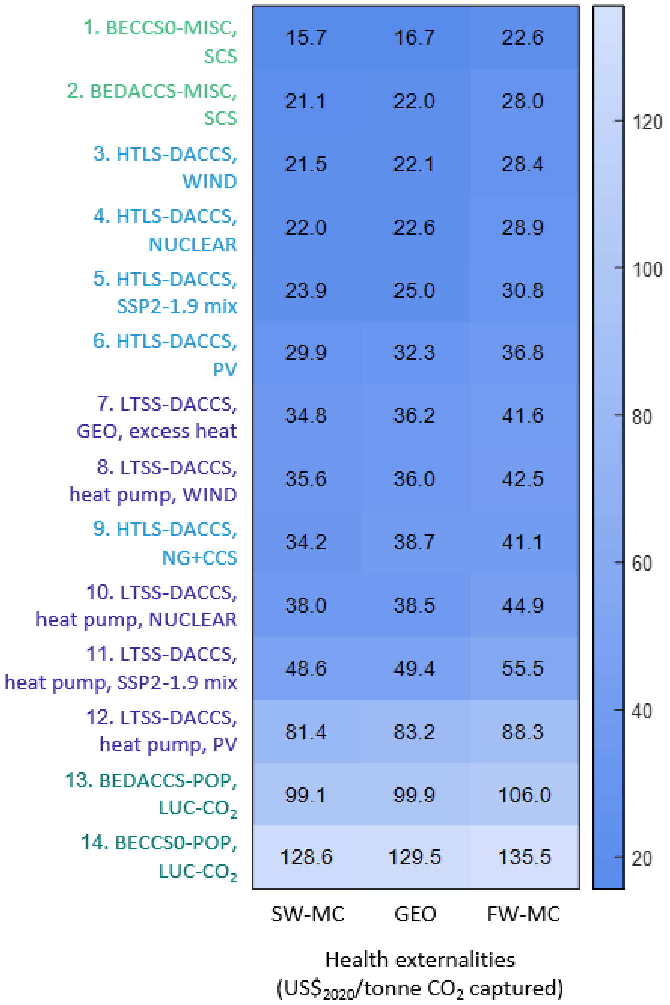

Supplementary Fig. 7. Health externalities of the scenarios based on geological sequestration at high pressure (GEO) and in situ mineralization with freshwater or seawater (FW-MC or SW-MC), expressed in US$$_{2020}$ per gross tonne CO_2 captured. Scenarios 1-16 comprise High-Temperature Liquid Sorbent (HTLS) and Low-Temperature Solid Sorbent (LTSS) DACCS – powered by natural gas with carbon capture and storage (NG+CCS), wind, solar photovoltaic (PV), nuclear, geothermal (GEO), or the global electricity mix deployed in the SSP2-1.9 marker scenario without NETs –, the basic BECCS scenarios (BECCS0) deploying *Miscanthus* (MISC) or poplar (POP) – assuming either Soil Carbon Sequestration (SCS) or land-use change (LUC) –, the hybrid BEDACCS configurations integrating BECCS0 and LTSS-DACCS, and the BECCS scenarios where CO_2 is mineralized ex situ (BECCS-EXSITU). The scenarios are ranked by the average health externalities, scenario 1 is the best.

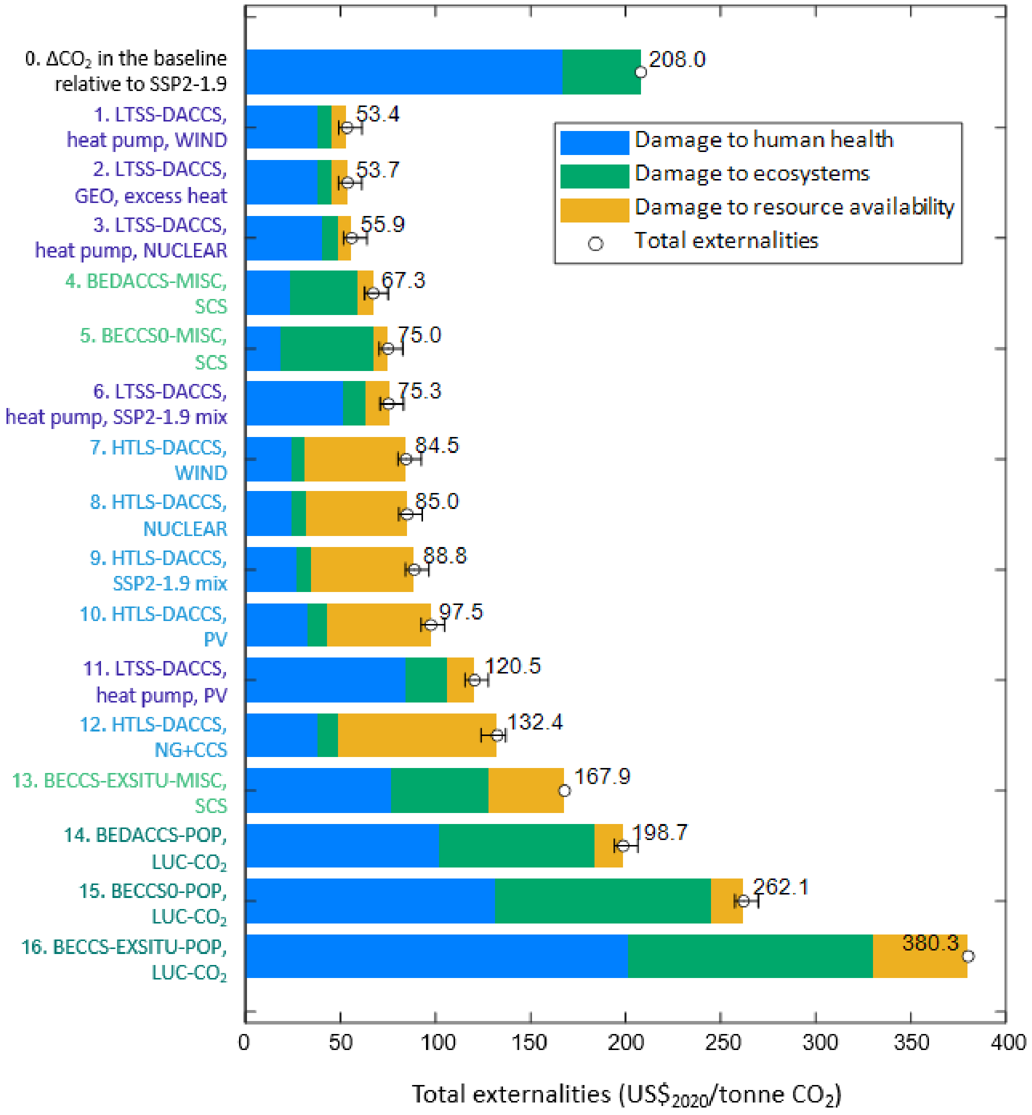

Supplementary Fig. 8. Total externalities, expressed in US$2020 per gross tonne CO_2 captured (scenarios 1-16) or emitted (scenario 0). Scenarios 1-16 comprise High-Temperature Liquid Sorbent (HTLS) and Low-Temperature Solid Sorbent (LTSS) DACCS – powered by natural gas with carbon capture and storage (NG+CCS), wind, solar photovoltaic (PV), nuclear, geothermal (GEO), or the global electricity mix deployed in the SSP2-1.9 marker scenario without NETs –, the basic BECCS scenarios (BECCS0) deploying *Miscanthus* (MISC) or poplar (POP) – assuming either Soil Carbon Sequestration (SCS) or land-use change (LUC) –, the hybrid BEDACCS configurations integrating BECCS0 and LTSS-DACCS, and the BECCS scenarios where CO_2 is mineralized ex situ (BECCS-EXSITU). Scenarios 1-16 are ranked by the total externalities, scenario 1 is the best. The black bars indicate the health impact range of the scenarios based on the in situ sequestration options, i.e., geological sequestration at high pressure and mineral carbonation with freshwater (upper bound) or seawater (lower bound).

1.2 CDR efficiency

Supplementary Table 1 shows the CO_2 removal (CDR) efficiency (η_{CO_2}) of the studied scenarios (quantified as the net kg CO_2 removed per kg CO_2 captured). These results were used to express the health and environmental impacts in terms of the functional unit.

Supplementary Table 1. CDR efficiency (η_{CO_2}, net kg CO_2 removed/kg CO_2 captured).

NET	Energy source	Geological sequestration	Freshwater mineralization	Seawater mineralization	Ex situ mineralization
LTSS-DACCS	GEO	0.9177	0.9212	0.9212	
	NUCLEAR	0.9483	0.9489	0.9489	
	PV	0.8500	0.8536	0.8536	
	WIND	0.9451	0.9458	0.9458	
	SSP2-1.9	0.9163	0.9179	0.9179	
HTLS-DACCS	NG+CCS	0.9240	0.9396	0.9396	
	NUCLEAR	0.9810	0.9819	0.9819	
	PV	0.9598	0.9645	0.9645	
	WIND	0.9804	0.9813	0.9813	
	SSP2-1.9	0.9741	0.9762	0.9762	
BECCS0-MISC		0.9307	0.9309	0.9309	
BECCS0-POP		0.8221	0.8223	0.8223	
BEDACCS-MISC		0.9443	0.9445	0.9445	
BEDACCS-POP		0.8664	0.8666	0.8666	
BECCS-EXSITU-MISC					0.8798
BECCS-EXSITU-POP					0.7527

1.3 Toxicity stressors

Supplementary Table 2 compiles the stressors contributing to over 95% of the carcinogenic and non-carcinogenic toxicity impacts across the studied scenarios.

Supplementary Table 2. Stressors responsible for >95% of human toxicity impacts.

Toxicity impact	Environmental flow	Emission compartment
Non-carcinogenic	Arsenic	Air
	Lead	Air
	Silver	Air
	Zinc	Air
	Barium	Soil
	Zinc	Soil
	Arsenic	Water
	Barium	Water
	Zinc	Water
Carcinogenic	Arsenic	Air
	Chromium VI	Air
	Nickel	Air
	Arsenic	Water
	Chromium VI	Water
	Nickel	Water

2. Life cycle models

This section describes the models used to compute the life cycle inventories of the assessed scenarios.

2.1 Direct Air Capture

In High-Temperature Liquid Sorbent Direct Air Capture (HTLS-DAC), atmospheric CO_2 is absorbed into a basic solution, which is regenerated with high-temperature heat. The HTLS-DAC model is based on *Carbon Engineering*'s DAC.[3] Here, natural gas supplies high-temperature heat, and the CO_2 derived from the combustion of natural gas is captured and sequestered. In configuration 1 of HTLS-DAC, natural gas is burnt in a turbine to generate electricity. The emissions data of natural gas combustion were taken from the literature.[4,5] The second HTLS-DAC configuration uses electricity from the grid or a renewable energy source. The HTLS-DAC process is based on two connected chemical loops; thus, the intermediate chemical products must be temporarily stored when intermittent energy sources (wind or solar photovoltaic) are used.

In Low-Temperature Solid Sorbent Direct Air Capture (LTSS-DAC), CO_2 is adsorbed onto a solid sorbent that is subsequently regenerated with low-temperature heat.[6] The energy consumption reported by *Climeworks*[7] was considered. Supplementary Table 3 shows the energy input of the studied DAC technologies (excluding the energy required to compress and sequester the CO_2, which varies with the storage options).

Supplementary Table 3. Energy consumption of DAC, excluding transport and storage (kWh/tonne captured CO_2).

	**HTLS-DAC Configuration 1**	**HTLS-DAC Configuration 2**	**LTSS-DAC Configuration 1**	**LTSS-DAC Configuration 2**
Electricity	0	366	650	1,561
Heat	2,447	1,458	2,000	0

We studied two LTSS-DAC configurations. In the first one, the source of low-temperature heat is the excess heat generated in the production of geothermal electricity. As Supplementary Table 3 shows, the needed electricity to heat ratio is about 1 to 3, whereas the ratio of electricity to excess heat that can be recovered in the modeled geothermal plant is approximately 1 to 5.[8]

We also considered the use of heat pumps based on working fluid R1234ze(E) to supply the low-temperature heat (configuration 2). We estimated the coefficient of performance (COP) with equation S1, where T_1 is the temperature of the heat source (ambient air at 288 K) and T_2 represents the temperature required to desorb the CO_2 (373 K). The efficiency of the heat pump (η_{hp}) is assumed to be 50%, which is within the typical range of efficiencies of industrial heat pumps.[9] With these data, we estimated a COP of 2.2, which leads to a total electricity consumption of 1,561 kWh/tonne CO_2 captured for this LTSS-DAC configuration.

$$COP = \frac{T_2}{T_2 - T_1} \cdot \eta_{hp} \qquad \text{(S1)}$$

The adsorbent consumption of the LTSS-DAC process is 7.5 kg/tonne.[10] The composition of the modeled adsorbent is 47.75% cellulose fiber, 47.75% polyethylenimine and 4.5% epoxy resin.[11] The production of polyethylenimine was modeled based on stoichiometric data and the typical yield (i.e., 87.5%) of the Wenker process.[12] The sodium sulfate generated as a byproduct of the

process is assumed to be landfilled, whereas the unreacted products are treated in a hazardous waste incineration plant. Lacking more accurate estimates, the energy consumption of the Wenker process was approximated based on the average energy demand of a large multi-product chemical plant, i.e., 3.2 MJ/kg (50% natural gas, 38% electricity and 12% steam).[13] The adsorbent is landfilled at the end of its lifetime.

The atmospheric water vapor retained in the adsorbent and subsequently desorbed with the low-temperature heat[14,15] is assumed to be released to the environment, without causing any impacts.

2.2 Bioenergy with Carbon Capture

The Bioenergy with Carbon Capture and Storage (BECCS) scenarios (BECCS0, BEDACCS and BECCS-EXSITU) are based on two configurations, BECCS0-MISC and BECCS0-POP. The biomass source *b* is Miscanthus and poplar in scenarios BECCS0-MISC and BECCS0-POP, respectively. We assumed that the land where the crops are planted was originally grassland. According to Qin et al.,[16] the median Soil Carbon Sequestration rate associated with planting Miscanthus and poplar in natural grasslands ($\mathrm{SCS_b}$) is 0.033 and -0.062 kg/ha/year within 0-100 cm of soil depth. We calculated the change in Soil Organic Carbon $\Delta\mathrm{SOC_b}$ associated with the production of 1 kg of biomass with equation S2.

$$\Delta SOC_b = \frac{SCS_b \cdot LO_b \cdot MW_{CO_2} \cdot D}{MW_C \cdot T} \quad \forall\, b \qquad \text{(S2)}$$

Assuming a transition period between equilibrium SOC values (D) of 20 years,[17] we prorated $\Delta\mathrm{SOC_b}$ over the evaluated time period T (71 years, between 2030 and 2100). $\mathrm{LO_b}$ represents the land occupation of Miscanthus and poplar (0.3750 and 0.3704 m^2·year/kg wet biomass), and $\mathrm{MW_{CO_2}}$ and $\mathrm{MW_C}$ denote the molecular weights of CO_2 and carbon. We estimated that $1.28 \cdot 10^{-2}$ kg CO_2 are sequestered per kg Miscanthus, and $2.37 \cdot 10^{-2}$ kg CO_2 are emitted per kg poplar (on a wet basis) due to the land transformation.

The inventories of *Miscanthus* and poplar were derived from [18] and [19], respectively. Supplementary Table 4 shows the main characteristics of the biomass. The BECCS technology is modeled based on the data provided by the IEA,[20] complemented with the biomass emission factors reported in [5]. The key differences between the technologies and the inputs and solid wastes of scenarios BECCS0-MISC and BECCS0-POP are displayed in Supplementary Tables 5 and 6. The energy balances of the BECCS scenarios were carried out with the data in Supplementary Table 7, derived from [20].

Supplementary Table 4. Biomass data.

	Miscanthus[18]	**Poplar**[19]
Biomass	Chopped grass	Wood chips
Moisture content (%)	42[21]	50
Carbon content (%, wet basis)	27.84	25.16
LHV (GJ/tonne, wet basis)	9.41[21]	7.32
Rotation period (years)	19.21	15
Land transformation (m^2/kg, wet basis)	$1.95 \cdot 10^{-2}$	$2.47 \cdot 10^{-2}$

Supplementary Table 5. Boiler characteristics.

	BECCS0-MISC	BECCS0-POP
Boiler	Circulating fluidized bed	Bubbling fluidized bed
Nominal capacity (MW_e)	250	75
Gross energy efficiency (%, LHV)	34.3	32.4

Supplementary Table 6. Inputs and solid wastes in the BECCS scenarios (kg/tonne biomass).

	BECCS0-MISC	BECCS0-POP
Bed material (limestone)	2.17	2.88
Boiler feedwater	34.06	43.27
Bottom ash	2.51	1.92
Fly ash	11.85	10.58

Supplementary Table 7. Data used to perform the energy balance of the BECCS scenarios.

	BECCS0-MISC	BECCS0-POP
Auxiliary electric load (kwh/tonne biomass)[a]	62.2	82.9
Ratio HP steam/biomass (tonne/GJ, LHV)	0.35	0.37
Ratio LP steam/HP steam (tonne/tonne)	0.73	0.81
Enthalpy HP steam (MJ/tonne)	3,469	3,459
Enthalpy LP steam (MJ/tonne)	2,988	2,723
Enthalpy LP steam, after CO_2 desorption (MJ/tonne)	573	577
Efficiency HP/MP turbine (MWh_e/MWh_{th})	0.23	0.21
Efficiency LP turbine (MWh_e/MWh_{th})	0.20	0.15

[a]Excluding CO_2 capture and compression.

Supplementary Table 8 shows the parameters of the CO_2 capture process. Supplementary Figure 9 depicts the carbon and energy flows of scenarios BECCS0, BEDACCS and BECCS-EXSITU.

Supplementary Table 8. Parameters describing the CO_2 capture from the flue gas stream.

CO_2 capture efficiency (%)		90
Energy input	Electricity for CO_2 capture (kWh/tonne CO_2 captured)[20]	28.7
	Desorption heat (GJ/tonne CO_2 captured)[20]	3.26
Material consumption	MEA make-up (kg/tonne CO_2 captured)[20]	2.5
	Water[b] (kg/tonne CO_2 captured)	5.84
	NaOH[c] (kg/tonne CO_2 captured)[22,23]	0.13
	Activated carbon[d] (kg/tonne CO_2 captured)[24]	0.075
Air emissions	NH_3 (kg/tonne CO_2 captured)[24]	0.35
	MEA (kg/tonne CO_2 captured)[25]	0.031
Waste	Solvent mixture to hazardous waste incineration (kg/tonne CO_2 captured)[23]	4.12

[b]30% weight MEA solution.[20]
[c]To recover MEA.
[d]To dry CO_2.

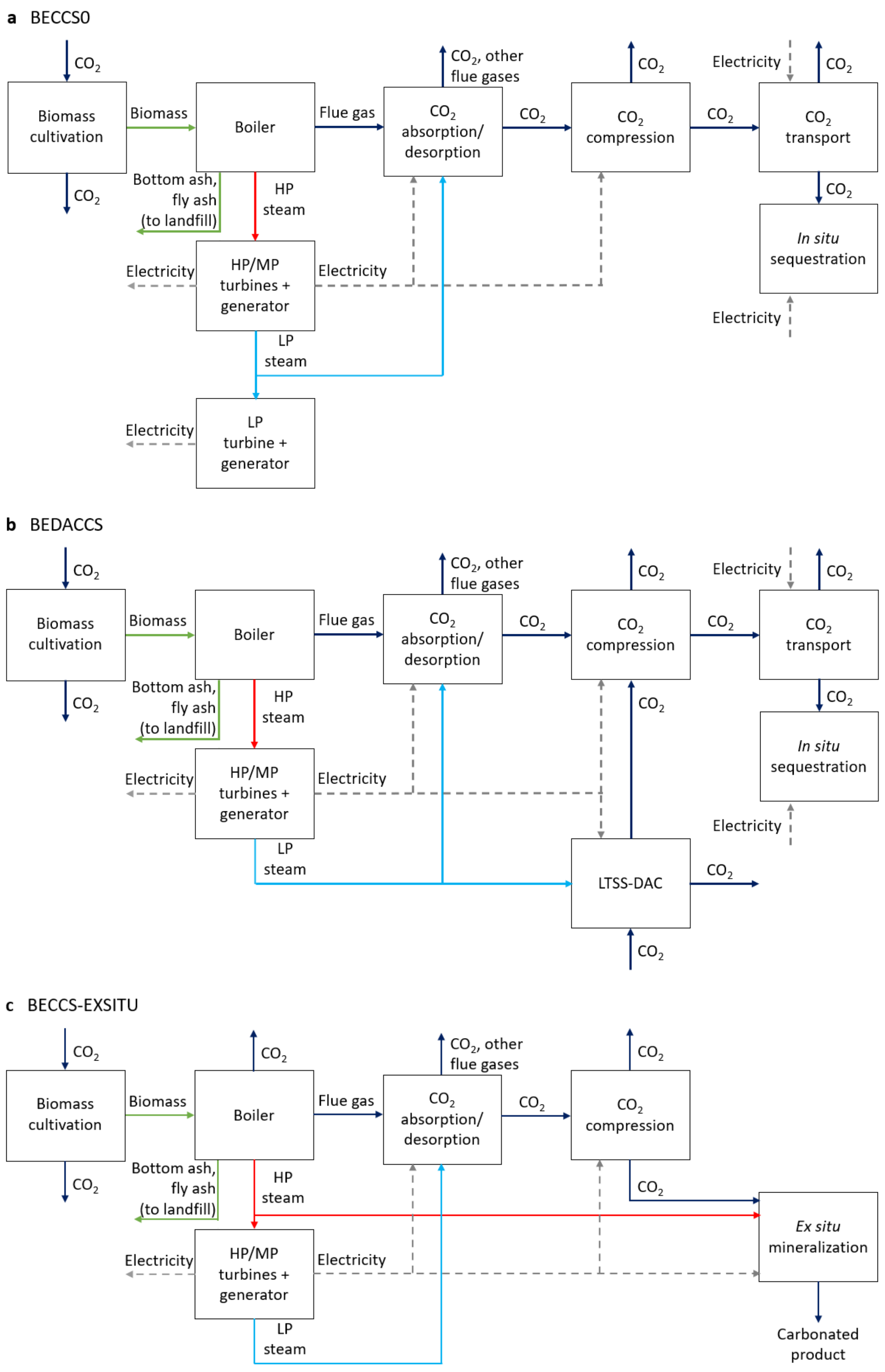

Supplementary Fig. 9. Carbon and energy flows between the foreground processes in the studied scenarios. a BECCS0. **b** BEDACCS. **c** BECCS-EXSITU.

The BECCS0 scenarios generate high-pressure (HP) and low-pressure (LP) steam. Part of the low-pressure (LP) steam is diverted from the turbine to supply the heat required to desorb the CO_2 generated in the biomass combustion from the monoethanolamine (MEA) solution. In the BEDACCS scenarios, the remaining LP steam (the fraction not used to desorb the CO_2 from the MEA solution) provides the low-temperature heat needed by the LTSS-DAC. Moreover, in the BECCS-EXSITU scenarios, part of the HP steam supplies the high-temperature heat required for the ex situ mineral carbonation. Since the energy content of the HP steam is fully exploited in the ex situ carbonation process,[26] there is insufficient LP steam to regenerate the MEA solution, and therefore 10-14% of the CO_2 generated in the biomass combustion process is directly released into the atmosphere in the BECCS-EXSITU scenarios. The electricity consumed in the MEA capture unit, the LTSS-DAC and the ex situ mineralization constitutes an additional energy penalty for the bioenergy plant.

2.3 Transport and sequestration

The in situ mineral carbonation processes using freshwater and seawater consume 27 and 31 m^3 of water per tonne CO_2 to dissolve the captured CO_2, respectively.[27] The electricity required to pump the water into the basalt formation (E_{pump}) was estimated with equation S3, where V is the specific water volume that must be pumped, the pump isentropic efficiency (η_{pump}) is 0.8, and the motor efficiency (η_{pm}) equals 0.9. The water pressure is increased from $P1_{H_2O}$ (1 bar) to the injection pressure ($P2_{H_2O}$), i.e., 1.5 bar.[28]

$$E_{pump} = \frac{V \cdot (P2_{H_2O} - P1_{H_2O})}{\eta_{pump} \cdot \eta_{pm}} \quad \text{(S3)}$$

The captured CO_2 must be compressed to be transported and injected into the geological reservoir (in the in situ sequestration options), or to react at the power plant with the magnesium extracted from the rocks (in the ex situ mineralization process). The CO_2 pressures required for the different sequestration options are compiled in Supplementary Table 9.

Supplementary Table 9. CO_2 discharge pressure.

**Sequestration option**	**CO_2 pressure, $P2_{CO_2}$ (bar)**
Geological sequestration	150[3]
In situ mineralization	25[29]
Ex situ mineralization	50[26]

The electricity required to compress the captured CO_2 (E_{comp}) was estimated with equation S4 using the parameters in Supplementary Table 10.[24]

$$E_{comp} = \frac{Z \cdot R \cdot T}{MW_{CO_2} \cdot \eta_{comp} \cdot \eta_{cm} \cdot 3600} \cdot \frac{N \cdot \gamma}{\gamma - 1} \cdot \left[\left(\frac{P2_{CO_2}}{P1_{CO_2}} \right)^{\frac{\gamma - 1}{N \cdot \gamma}} - 1 \right] \quad \text{(S4)}$$

Supplementary Table 10. Parameters to estimate the energy consumption of CO_2 compression.

Parameter		Value
Z	Compressibility factor	0.9942
R	Universal gas constant	8.3144 kJ/kmol/K
T	Suction temperature	313.15 K
γ	Specific heat ratio	1.2938
N	Number of compressor stages	Initial compression, N=4,<br>Recompression, N=2
η_{comp}	Compressor isentropic efficiency	0.8
η_{cm}	Motor efficiency	0.9
$P1_{CO_2}$	Suction pressure	1 bar

Due to the pressure drop that occurs during the CO_2 transportation process (0.06 bar/km),[24] the transported CO_2 must be recompressed prior to injection. The fugitive CO_2 emissions associated with compression and transport are $2.90\cdot10^{-6}$ tonne/kWh and $7.33\cdot10^{-8}$ tonne/km/ton,[24] respectively.

Given the modular characteristics of DAC,[3,6] we assumed that the DACCS and BEDACCS plants are located next to the sequestration site regardless of the sequestration configuration. In situ mineralization requires a lower CO_2 injection pressure than the conventional geological sequestration; consequently, its electricity consumption is lower. However, if the CO_2 is transported, it must be compressed at a higher pressure than that required for the in situ mineralization. Therefore, we assume that the bioenergy plants are located next to the sequestration site in the scenarios deploying in situ and ex situ mineralization, and 400 km away from the sequestration point (CO_2 transported at 150 bar by pipeline) in the configurations based on geological storage. We consider a conservative distance of 200 km (by road) from the biomass cultivation site to the power plant.

In the BECCS-EXSITU scenarios, the products of the ex situ mineral carbonation (magnesium carbonate and unreacted magnesium hydroxide) are used to backfill the mine from which the rock (serpentine) used in the ex situ mineralization process is extracted.

We did not consider any commercial applications for the product of the ex situ mineralization because, given the large scale at which the mineralization process is deployed, the chemical market is unlikely to be able to absorb it.[30] On the contrary, we expanded the system boundaries to consider the application of the byproducts of the carbonation process, assuming that the iron precipitated as FeOOH replaces the iron contained in beneficiated iron ore (65% iron), and that the produced silicon dioxide and the unreacted rocks replace sand used as an inert filler.

The mass balance of the ex situ mineralization process was carried out with the data derived from [26,31]. We estimated that 4.46 tonne rock are required to sequester 1 tonne CO_2, based on the best magnesium conversion efficiency reported to date (56%),[32] and the following rock composition: 84% serpentine, 13% FeO and 3% $CaSiO_3$.[31] Moreover, we assumed that the impacts of the rock mining activities are the same as those associated with the operation of an iron mine.

Based on the data reported by [26,31,33], a heat requirement of 4.95 GJ/tonne CO_2 was estimated for the ex situ mineralization process with the studied magnesium conversion efficiency. The electricity consumed to crush and grind the rocks to 100 µm[34] is 13.40 kWh/tonne.[35]

2.4 Electricity mix

We estimated the average contribution of energy source *es* to the global electricity mix deployed in the SSP2-1.9 marker scenario between 2030 and 2100 ($\mathrm{AVMIX_{es}}$) with equation S5, where $\mathrm{MIX_{es,t}}$ represents the fraction of energy source es in the electricity mix of year t, and $\mathrm{CDR_t}$, the CDR rate.

$$AVMIX_{es} = \frac{\sum_{t\in T} MIX_{es,t} \cdot CDR_t}{\sum_{t\in T} CDR_t} \quad \forall\, es \tag{Eq. S5}$$

Supplementary Table 11 shows the CDR rates and the contributions of the deployed energy sources to the electricity mix across the studied period.[36] In the HTLS- and LTSS-DACCS scenarios, we assume that BECCS is not deployed. Moreover, the electricity generated in the BECCS scenarios replaces the electricity produced with other technologies. Therefore, $\mathrm{AVMIX_{es}}$ does not include the contribution of BECCS to the mix, i.e., $\mathrm{AVMIX_{es=BECCS}} = 0$. The shares of each energy source are re-scaled to sum up 100% after removing the BECCS contribution.

Supplementary Table 11. CDR rates and composition of the energy mix between 2030 and 2100.

	**2030**	**2040**	**2050**	**2060**	**2070**	**2080**	**2090**	**2100**
CDR_t (Gtonne)	0.07	0.36	1.31	3.29	7.21	10.72	11.63	12.36
$MIX_{es,t}$ (%)								
Oil	0.53	0.17	0.04	0.01	0.00	0.00	0.00	0.00
Coal w/o CCS	2.89	0.00	0.00	0.00	0.00	0.00	0.00	0.00
Coal w/CCS	1.20	1.19	0.45	0.22	0.09	0.02	0.00	0.00
Gas w/o CCS	26.49	5.64	1.30	0.90	0.39	0.10	0.00	0.00
Gas w/CCS	4.39	12.20	9.81	5.12	2.21	0.55	0.00	0.00
Biomass w/o CCS	0.67	0.41	0.31	0.33	0.17	0.06	0.04	0.03
Geothermal	0.67	1.47	1.61	1.61	1.63	1.49	1.39	1.27
Hydro	18.93	17.61	14.00	11.51	10.00	9.11	8.40	7.68
Nuclear[e]	16.52	19.49	25.88	32.55	32.43	29.66	24.02	15.53
Solar PV	6.84	10.12	13.88	15.87	18.97	20.54	23.48	28.08
Solar thermal	0.07	0.23	0.46	0.78	1.25	1.97	2.95	4.26
Onshore wind	20.10	29.52	29.14	27.95	29.40	32.64	34.41	36.09
Offshore wind	0.70	1.95	3.11	3.14	3.46	3.87	5.31	7.06

[e]Pressure water reactor.

2.5 Potential deployment constraints

Supplementary Table 12 compares the energy required to remove 5.9 net Gtonne/year CO_2 (scenario SSP2-1.9) via LTSS-DAC coupled to geological sequestration – the DACCS configuration with the highest consumption of renewable energy – to the ranges of global technical potentials estimated for renewable energy sources.[37] The required energy inputs are below the maximum technical potentials estimated for the assessed energy sources, confirming the technical feasibility of the proposed configurations.

Supplementary Table 12. Range of global technical potentials of renewable energy sources compared to the energy consumed to remove 5.9 net Gtonne/year CO_2 with LTSS-DAC coupled to geological sequestration (EJ/year) in scenario SSP2-1.9.

		**Electricity (EJ/year)**		**Heat (EJ/year)**
	**Geothermal**	**Wind**	**Solar photovoltaic**	**Geothermal**
Global technical potentials	118-1,109	85-580	315-9,967[f]	10-312
LTSS-DACCS (Configuration 1)	18.00			46.05
LTSS-DACCS (Configuration 2)[g]		37.85	42.08	

[f]Assuming a 20% energy conversion efficiency.[38]

[g]The electricity consumed by LTSS-DACCS powered by wind and solar photovoltaic to attain the same functional unit diverges due to the different CO_2 emissions associated with each electricity source.

We also evaluated the total area of grassland needed to fulfill the functional unit in scenarios BECCS-EXSITU-MISC and BECCS-EXSITU-POP – those with the largest biomass consumption – against the current global area of natural and semi-natural grasslands ($6.7 \cdot 10^6$ km^2).[39] We estimated that scenario BECCS-EXSITU-MISC and BECCS-EXSITU-POP would require 2.1 and 3.9% of the current global grassland area, respectively.

Finally, it has been estimated that the global reserves of serpentine are sufficient to sequester global CO_2 emissions,[40] whereas basaltic rocks are widely abundant on the Earth's surface.[41] Therefore, rock availability will not act as a bottleneck for the large-scale deployment of the mineral carbonation processes.

3. Supplementary methods

Here we describe the methods used to evaluate the human health and Earth-system impacts, and the externalities.

3.1 Human health impact assessment

3.1.1 Health damage factors of CO_2 emissions

Supplementary Table 13 compiles the breakdown of the damage factors used to calculate the climate-related health impacts by health risk and region for SSP2.[42] The countries and territories comprised within each region are displayed in Supplementary Table 14.

Supplementary Table 13. Damage factors for SSP2 (DALY/kg CO_2).

	Undernutrition	Malaria	Coastal floods	Diarrhea	Heat stress	Dengue
North Africa/Middle East	$3.90\cdot10^{-8}$	$2.00\cdot10^{-9}$	$9.00\cdot10^{-11}$	$2.20\cdot10^{-9}$	$1.80\cdot10^{-9}$	$1.00\cdot10^{-10}$
Sub-Saharan Africa	$4.93\cdot10^{-7}$	$2.00\cdot10^{-7}$	$2.02\cdot10^{-9}$	$4.66\cdot10^{-8}$	$3.58\cdot10^{-9}$	$1.03\cdot10^{-11}$
South Asia	$3.50\cdot10^{-7}$	$5.00\cdot10^{-9}$	$6.00\cdot10^{-8}$	$2.50\cdot10^{-8}$	$7.20\cdot10^{-9}$	$6.00\cdot10^{-11}$
Southeast Asia	$6.50\cdot10^{-8}$	$5.00\cdot10^{-9}$	$5.00\cdot10^{-8}$	$1.20\cdot10^{-9}$	$1.50\cdot10^{-9}$	$6.00\cdot10^{-14}$
Central/East Asia	$3.73\cdot10^{-8}$	$9.00\cdot10^{-13}$	$1.01\cdot10^{-8}$	$4.82\cdot10^{-10}$	$4.55\cdot10^{-9}$	$-2.50\cdot10^{-12}$
Eastern Europe/Russia	0.00	0.00	$7.00\cdot10^{-11}$	$5.20\cdot10^{-12}$	$8.90\cdot10^{-10}$	0.00
West/Central Europe	0.00	0.00	$2.40\cdot10^{-10}$	$4.16\cdot10^{-12}$	$1.91\cdot10^{-9}$	0.00
North America	0.00	$4.00\cdot10^{-13}$	0.00	$2.20\cdot10^{-12}$	$1.80\cdot10^{-9}$	$-1.00\cdot10^{-12}$
Caribbean	0.00	$3.00\cdot10^{-9}$	0.00	$6.80\cdot10^{-11}$	$7.90\cdot10^{-11}$	$-3.00\cdot10^{-12}$
Latin America	$2.16\cdot10^{-8}$	$1.10\cdot10^{-10}$	$3.00\cdot10^{-11}$	$2.01\cdot10^{-10}$	$1.58\cdot10^{-9}$	$-7.85\cdot10^{-11}$
Oceania	0.00	$3.08\cdot10^{-10}$	$6.40\cdot10^{-10}$	$2.90\cdot10^{-11}$	$9.60\cdot10^{-11}$	$-2.00\cdot10^{-11}$

Supplementary Table 14. List of countries and territories within the aggregated regions.

Regions	Countries/Territories
North Africa/ Middle East	Algeria, Bahrain, Egypt, Iran, Iraq, Jordan, Kuwait, Lebanon, Libya, Morocco, Palestine, Oman, Qatar, Saudi Arabia, Syrian Arab Republic, State of Palestine, Tunisia, Turkey, United Arab Emirates, Western Sahara, Yemen
Sub-Saharan Africa	Angola, Central African Republic, Congo, Democratic Republic of the Congo, Equatorial Guinea, Gabon Burundi, Comoros, Djibouti, Eritrea, Ethiopia, Kenya, Madagascar, Malawi, Mayotte, Mozambique, Rwanda, Somalia, Sudan, Uganda, United Republic of Tanzania, Zambia, Botswana, Lesotho, Namibia, South Africa, Swaziland, Zimbabwe, Benin, Burkina Faso, Cameroon, Cape Verde, Chad, Cote d'Ivoire, Gambia, Ghana, Guinea, Guinea-Bissau, Liberia, Mali, Mauritania, Niger, Nigeria, Saint Helena, Sao Tome and Principe, Senegal, Sierra Leone, Togo
South Asia	Afghanistan, Bangladesh, Bhutan, India, Nepal Pakistan
Southeast Asia	Cambodia, Christmas Island, Cocos Islands, Indonesia, Lao People's Democratic Republic, Malaysia, Maldives, Mauritius, Myanmar, Philippines, Reunion, Seychelles, Sri Lanka, Thailand, Timor-Leste, Viet Nam
Central/East Asia	Brunei Darussalan, Japan, Republic of Korea, Singapore, Armenia, Azerbaijan, Georgia, Kazakhstan, Kyrgyzstan, Mongolia, Tajikistan, Turkmenistan, Uzbekistan, China, Democratic People's Republic of Korea
Eastern Europe/ Russia	Belarus, Estonia, Latvia, Lithuania, Republic of Moldova, Russia, Ukraine
Western/ Central Europe	Albania, Bosnia and Herzegovina, Bulgaria, Croatia, Czech Republic, Hungary, Montenegro, Poland, Romania, Serbia, Slovakia, Slovenia, Republic of North Macedonia, Akrotiri and Dhekelia, Åland Islands, Andorra, Austria, Belgium, Channel Islands, Cyprus, Denmark, Faroe Islands, Finland, France, Germany, Gibraltar, Greece, Greenland, Guernsey, Holy See, Iceland, Ireland, Isle of Man, Israel, Italy, Jersey, Liechtenstein, Luxembourg, Malta, Monaco, Netherlands, Norway, Portugal, San Marino, Spain, Svalbard, Sweden, Switzerland, United Kingdom of Great Britain and Northern Ireland
North America	Canada, Saint Pierre et Miquelon, United States of America
Caribbean	Anguilla, Antigua and Barbuda, Aruba, Bahamas, Barbados, Belize, Bermuda, British Virgin Islands, Cayman Islands, Cuba, Dominica, Dominican Republic, French Guiana, Grenada, Guadeloupe, Guyana, Haiti, Jamaica, Martinique, Montserrat, Netherlands Antilles, Puerto Rico, Saint Barthelemy, Saint Kitts and Nevis, Saint Lucia, Saint Martin, Saint Vincent and the Grenadines, Suriname, Trinidad and Tobago, Turks and Caicos Islands, US Virgin Islands
Latin America	Plurinational State of Bolivia, Ecuador, Peru, Colombia, Costa Rica, El Salvador, Guatemala, Honduras, Mexico, Nicaragua, Panama, Venezuela, Argentina, Chile, Falkland Islands (Islas Malvinas), Uruguay, Brazil, Paraguay
Oceania	America Samoa, Cook Islands, Fiji, French Polynesia, Guam, Kiribati, Marshall Islands, Federated States of Micronesia, Nauru, New Caledonia, Niue, Norfolk Island, Northern Mariana Islands, Palau, Papua New Guinea, Pitcairn, Samoa, Solomon Islands, Tokelau, Tonga, Tuvalu, Vanuatu, Wallis and Fortuna Islands

3.2 Estimation of externalities

We monetized the human health impacts linked to the capture of 1 tonne CO_2 by applying the conversion factor proposed by Weidema (1 DALY = 74,000 $€_{2003}$) –[43] to the non-climate and climate-related health impacts, which were estimated with the ReCiPe 2016 endpoint method[44] (Hierarchist perspective) and the health damage factors provided by Tang et al.[42] for SSP2, respectively.

We also estimated the externalities associated with the damage to the other areas of protection differentiated within the ReCiPe 2016 endpoint method, i.e., ecosystem quality and resource availability. We applied the following equivalence: 1 lost species·year = $9.5 \cdot 10^6$ $€_{2003}$ to translate the damage to ecosystems calculated with the ReCiPe Hierarchist perspective into monetary units.[43] The ReCiPe 2016 endpoint method expresses damage to resource availability in $US\$_{2013}$. We used the currency conversion factors and GDP deflators found in [45,46] to express the monetary units in $US\$_{2020}$.

The externalities were compared to the levelized CO_2 cost of scaled-up HTLS-DACCS (181-249 US$/tonne for configuration 1 and 121-175 US$/tonne for configuration 2)[3] and combustion BECCS (134-188 US$/tonne).[20] The levelized CO_2 cost of BECCS was estimated by subtracting the electricity revenues – assuming an electricity selling price of 130 €/MWh[47] – from the total BECCS costs provided in [20] and adjusted for inflation.[46] The levelized CO_2 cost of LTSS-DACCS is currently about 600 US$/tonne[48], although *Climeworks* expects the cost to drop to approximately 100 US$/tonne by 2030.[6]

3.3 Earth-system impact assessment

To conduct the Earth-system impact assessment, we adjusted some of the characterization factors described in [49], as Supplementary Table 15 shows.

Supplementary Table 15. Refinements of the characterization factors developed by Ryberg et al.[49]

Earth-system process	Control variable	Environmental flow	Emission compartment	Characterization factor	Change
Climate change	Atmospheric CO_2 concentration	CO_2, land transformation	Air	$2.69 \cdot 10^{-11}$ ppm·year/kg	Refined characterization factor
Climate change	Energy imbalance at top-of-atmosphere	CO_2, land transformation	Air	$3.53 \cdot 10^{-13}$ W·year/m^2/kg	Refined characterization factor
Ocean acidification	CO_3^{2-} concentration, average global surface ocean saturation state with respect to aragonite	CO_2, land transformation	Air	$8.22 \cdot 10^{-14}$ mol·year/kg	Refined characterization factor
Biogeochemical flows	*Global, N cycle:* industrial and intentional biological fixation of N	–	–	–	Characterization factors omitted
Biogeochemical flows	*Global, P cycle:* P flow from freshwater systems into the ocean	PO_4^{3-}	Freshwater	$2.81 \cdot 10^{-10}$ Tg·year/kg/year	Characterization factor added
Stratospheric ozone depletion	Stratospheric ozone concentration	N_2O	Air	$1.41 \cdot 10^{-10}$ DU·year/kg	Characterization factor added

To calculate the characterization factors used to estimate the climate change and ocean acidification impacts of the *CO_2 from land transformation* environmental flow, Ryberg et al.[49] divided the CO_2 characterization factors by the time horizon (300 years). They did that under the assumption that this elementary flow appears in the inventories as a pulse emission that must be annualized. However, the CO_2 emissions due to LUC that occur in the foreground systems are already annualized in our model (equation S2). Likewise, the CO_2 emissions from land transformation provided by the Ecoinvent processes are calculated based on the annual change in Soil Organic Carbon, following the IPCC recommendations.[17] Hence, we applied the characterization factors of CO_2 to the *CO_2 from land transformation* environmental flow, consistently with other impact assessment methods (e.g., IPCC 2013,[50] ReCiPe 2016[44]).

We defined a characterization factor for PO_4^{3-} – calculated as the product of the P characterization factor (freshwater emission compartment) and the mass fraction of P in PO_4^{3-} – to estimate the impact on the P flow. We also defined a characterization factor to account for the contribution of N_2O to stratospheric ozone depletion, as suggested in Algunaibet et al.[51]

The characterization factors developed by Ryberg et al.[49] to estimate the impacts on the N flow require special attention. First, the life cycle assessment practitioner must select only one of the main environmental compartments where emissions occur to avoid double accounting, since emissions of N to different compartments can be due to the same amount of N fixed. Second,

these characterization factors are calculated via inverse modeling and based on global parameters. However, they should be site-specific, depending on local conditions such as soil properties.

To produce more accurate estimates, we directly calculated the total amount of N fixed in the following Ecoinvent activities, which are part of the supply chains of the assessed systems:

Ammonium nitrate, as N|ammonium nitrate production
Ammonium nitrate, as N|calcium nitrate production
Ammonium sulfate, as N|ammonium sulfate production
Nitrogen fertilizer, as N|ammonium nitrate phosphate production
Nitrogen fertilizer, as N|calcium ammonium nitrate production
Nitrogen fertilizer, as N|diammonium phosphate production
Nitrogen fertilizer, as N|monoammonium phosphate production
Nitrogen fertilizer, as N|urea ammonium nitrate production
Urea, as N|production
Nitrogen fertilizer, as N|nutrient supply from ammonium chloride
Nitrogen fertilizer, as N|nutrient supply from calcium nitrate
Nitrogen fertilizer, as N|nutrient supply from potassium nitrate

These activities were selected in accordance with the definition of the N Planetary Boundary (PB) control variable, i.e., the application rate of intentionally fixed N in the agricultural system.[2] Therefore, the activities fixing N for non-agricultural purposes must be excluded from the analysis of the impacts on the N flow. In our study, we only omitted the N linked to the ammonium sulfate used in the ex situ mineral carbonation process from the above-mentioned list of activities. Hence, this approach – like the method developed by Ryberg et al.[49] – might be overestimating the impacts, because some of these products could have non-agricultural applications in the background processes, e.g., in mining activities.

Supplementary Table 16 compiles the values of the PBs, the natural background (NB) and the full Safe Operating Space (SOS) of the assessed Earth-system processes, obtained from [2,49]. According to Bouwman et al.[52] – the source used by de Vries et al.[53] to estimate the N PB –, 68% of the intentionally fixed N in the agricultural system is associated with industrial fertilizers, and 32% is biologically fixed. Since our models do not account for the biologically fixed N, the value of the N PB provided by Steffen et al.[2] was multiplied by 0.68 to define the N PB for industrial fertilizers.

Supplementary Table 16. Data used to estimate the impacts relative to the SOS size.

Earth-system process / Control variable	PB	NB	Full SOS	Unit
Climate change / CO_2 concentration	350.00	278.00	72.00	ppm
Climate change / Energy imbalance	1.00	0.00	1.00	w/m^2
Terrestrial biosphere integrity	10.00	0.00	10.00	%
Ocean acidification	2.75	3.44	0.69	mol
Biogeochemical flows / N, global	42.18	0.00	42.18	Tg N/year
Biogeochemical flows / P, global	11.00	1.10	9.90	Tg P/year
Freshwater use / Global	4000.00	0.00	4000.00	km^3/year
Stratospheric ozone depletion	275.50	290.00	14.50	DU
Land-system change / Global	75.00	100.00	25.00	%

4. Assumptions and limitations

Here we list the main assumptions and limitations of our life cycle models and the impact assessment methods.

4.1 Life cycle models

In accordance with the recommended guidelines for prospective life cycle assessment studies, emerging technologies in early development stages modeled at a large-scale deployment level should avoid temporal mismatches between the background and foreground activities.[54] Background activities are described with homogenous data based on average market values, while foreground activities are specific to the studied system.[55] However, modeling all the background activities and the structural market changes that may result from deploying Negative Emissions Technologies (NETs) at a large scale would require making additional assumptions regarding the evolution of future technologies, thereby resulting in more pronounced uncertainties. Hence, the background activities of our scenarios, including electricity generation, rely on the current average market mixes.

We compared the health and environmental impacts of the 2018 global electricity mix (composition taken from the IEA[56]) and the future electricity mix used here for the foreground activities (Supplementary Tables 17 and 18) to assess how changing the electricity mix in the background activities would affect our analysis. We found that, although increasing the share of nuclear energy would lead to higher health impacts linked to ionizing radiation, the overall health and environmental impacts of the future decarbonized mix would be lower. Accordingly, our results represent conservative estimates.

Another limitation of the study is that we did not conduct a regionalized analysis. However, the health impacts linked to ozone formation, water consumption, fine particulate matter formation and toxicity vary across different regions. Furthermore, additional trade-offs between the PBs operating at regional scales (the regional phosphorus PB, the land-system change PBs identified for different biomes, the basin-scale PB for freshwater withdrawal, and the regional atmospheric aerosol loading PBs)[2] could arise.

Supplementary Table 17. Health impacts of generating of 1 PWh/year of electricity (DALY/year).

Environmental mechanism	2018 mix	Future mix
Global warming	8.36E+05	4.87E+04
Stratospheric ozone depletion	9.96E+01	1.66E+01
**Ionizing radiation**	**6.24E+02**	**1.49E+03**
Ozone formation	1.46E+03	8.47E+01
Fine particulate matter formation	6.90E+05	4.60E+04
Human carcinogenic toxicity	6.69E+04	1.60E+04
Human non-carcinogenic toxicity	1.69E+05	2.51E+04
Water consumption	1.35E+04	2.71E+03
Total health impacts	1.78E+06	1.40E+05

Supplementary Table 18. Environmental impacts of generating of 1 PWh/year of electricity (SOS%).

Earth-system process	2018 mix	Future mix
Climate change / CO_2 concentration	20.55	1.10
Climate change / Energy imbalance	19.54	1.07
Ocean acidification	6.57	0.35
Terrestrial biosphere integrity	1.34	0.15
Biogeochemical flows / N, global	0.29	1.68E-02
Biogeochemical flows / P, global	2.39E-03	1.65E-03
Freshwater use / Global	0.17	0.02
Stratospheric ozone depletion	1.59E-02	2.66E-03
Land-system change / Global	1.95E-04	2.21E-05

4.2 Impact assessment method

We only quantified the climate-sensitive health impacts related to a subset of health risks, namely undernutrition, malaria, coastal floods, diarrhea, heat stress and dengue.[42] On the other hand, the method used to estimate the impacts on the terrestrial biosphere[57] only considers two main stressors, namely greenhouse gas emissions and land use.[58,59] Therefore, the averted climate-sensitive health effects and the impacts on the terrestrial biosphere could be higher. Conversely, we could be overestimating the impact on the N flow Earth-system process because some of the products responsible for N fixation in the background system may have non-agricultural applications and thus may not affect the N PB control variable.

The damage to the biosphere integrity and the health impacts are aggregated over a 100-year time horizon, following the consensus scientific models.[44] Nevertheless, the life cycle impact assessment method used to quantify the climate change and ocean acidification impacts based on the PB framework considers a 300-year time horizon because the model used to derive the climate change characterization factors attains the stabilization of the atmospheric CO_2 concentration at a level similar to that of the climate change PB over a 300-year period.[49] Thus, shorter time horizons would lead to results that do not reflect the level of greenhouse gas emissions that allows humanity to operate within the SOS of the climate change Earth-system process.

5. Supplementary references

1. World Health Organization. *Global Health Estimates 2019: Disease burden by Cause, Age, Sex, by Country and by Region, 2000-2019.* https://www.who.int/data/gho/data/themes/mortality-and-global-health-estimates/global-health-estimates-leading-causes-of-dalys (2020).
2. Steffen, W. *et al.* Planetary boundaries: Guiding human development on a changing planet. *Science.* **347**, (2015).
3. Keith, D. W., Holmes, G., St. Angelo, D. & Heidel, K. A Process for Capturing CO_2 from the Atmosphere. *Joule* **2**, 1573–1594 (2018).
4. Gómez, D. R. et al. Chapter 2. Stationary combustion. In *IPCC guidelines for national greenhouse gas inventories* (2006).
5. European Environment Agency. Energy industries. In *EMEP/EEA air pollutant emission inventory guidebook 2019* (2019).
6. Beuttler, C., Charles, L. & Wurzbacher, J. The Role of Direct Air Capture in Mitigation of Anthropogenic Greenhouse Gas Emissions. *Front. Clim.* **1**, (2019).
7. Climeworks. https://climeworks.com/faq-about-direct-air-capture?gclid=Cj0KCQjwg8n5BRCdARIsALxKb952fWFAdoyzPfbUqEy1CdZxJ_uDiSBIcQcu2yQ61cuuCQgZ2mqwODQaApPXEALw_wcB [last accessed: 15/03/2021].
8. Hirschberg, S., Wiemer, S. & Burgherr, P. *Energy from the Earth. Deep geothermal as a resource for the future?* (2015).
9. Arpagaus, C., Bless, F., Uhlmann, M., Schiffmann, J. & Bertsch, S. S. High temperature heat pumps: Market overview, state of the art, research status, refrigerants, and application potentials. *Energy* **152**, 985–1010 (2018).
10. Deutz, S. & Bardow, A. Life-cycle assessment of an industrial direct air capture process based on temperature–vacuum swing adsorption. *Nat. Energy* **6**, 203–213 (2021).
11. Gebald, C., Wurzbacher, J.A., Steinfeld, A. Amine containing fibrous structure for adsorption of CO_2 from atmospheric air. WO 2010/091831 Al. (2010).
12. Steuerle, U., Feuerhake, R. Aziridines. *Ullmann's encyclopedia of industrial chemistry* vol. 4, 515–522 (2012).
13. Althaus, H. *et al. Life cycle inventories of chemicals. Data v2.0. Ecoinvent* (2007).
14. Wurzbacher, J. A., Gebald, C., Piatkowski, N. & Steinfeld, A. Concurrent Separation of CO_2 and H_2O from Air by a Temperature-Vacuum Swing Adsorption/Desorption Cycle. *Environ. Sci. Technol.* **46**, 9191–9198 (2012).
15. Gebald, C., Wurzbacher, J. A., Borgschulte, A., Zimmermann, T. & Steinfeld, A. Single-component and binary CO_2 and H_2O adsorption of amine-functionalized cellulose. *Environ. Sci. Technol.* **48**, 2497–2504 (2014).
16. Qin, Z., Dunn, J. B., Kwon, H., Mueller, S. & Wander, M. M. Soil carbon sequestration and land use change associated with biofuel production: Empirical evidence. *GCB Bioenergy* **8**, 66–80 (2016).
17. Aalde, H. *et al. Chapter 2. Generic methodologies applicable to multiple land-use categories. In: IPCC Guidelines for National Greenhouse Gas Inventories*. *IPCC Guidelines for National Greenhouse Gas Inventories* (2006).
18. Wernet, G. *et al.* The ecoinvent database version 3 (part I): overview and methodology. *Int. J. Life Cycle Assess.* **21**, 1218–1230 (2016).
19. Peters, J. F., Iribarren, D. & Dufour, J. Biomass pyrolysis for biochar or energy applications? A life cycle assessment. *Environ. Sci. Technol.* **49**, 5195–5202 (2015).
20. IEA GHG. *Biomass CCS Study*. (2009).
21. Phyllis2 database. https://phyllis.nl/Browse/Standard/ECN-Phyllis#miscanthus [last accessed: 09.12.21].
22. Rao, A. B. & Rubin, E. S. A technical, economic, and environmental assessment of amine-based CO_2 capture technology for power plant greenhouse gas control. *Environ.*

Sci. Technol. **36**, 4467–4475 (2002).
23. Rao, A. B., Rubin, E. S. & Berkenpas, M. B. *An integrated modeling framework for carbon management technologies. Volume 1 – Technical Documentation: Amine-Based CO_2 Capture and Storage Systems for Fossil Fuel Power Plant*. (2004).
24. Koornneef, J., van Keulen, T., Faaij, A. & Turkenburg, W. Life cycle assessment of a pulverized coal power plant with post-combustion capture, transport and storage of CO_2. *Int. J. Greenh. Gas Control* **2**, 448–467 (2008).
25. International Energy Agency Greenhouse Gas R&D Programme. *Environmental Impact of Solvent Scrubbing of CO_2*. (2006).
26. Romão, I., Nduagu, E., Fagerlund, J., Gando-Ferreira, L. M. & Zevenhoven, R. CO_2 fixation using magnesium silicate minerals. Part 2: Energy efficiency and integration with iron-and steelmaking. *Energy* **41**, 203–211 (2012).
27. Gislason, S. R. *et al.* Mineral sequestration of carbon dioxide in basalt: A pre-injection overview of the CarbFix project. *Int. J. Greenh. Gas Control* **4**, 537–545 (2010).
28. Sigfusson, B. *et al.* Solving the carbon-dioxide buoyancy challenge: The design and field testing of a dissolved CO_2 injection system. *Int. J. Greenh. Gas Control* **37**, 213–219 (2015).
29. Snæbjörnsdóttir, S. Ó. *et al.* Carbon dioxide storage through mineral carbonation. *Nat. Rev. Earth Environ.* **1**, 90–102 (2020).
30. Kirchofer, A., Brandt, A., Krevor, S., Prigiobbe, V. & Wilcox, J. Impact of alkalinity sources on the life-cycle energy efficiency of mineral carbonation technologies. *Energy Environ. Sci.* **5**, 8631–8641 (2012).
31. Fagerlund, J., Nduagu, E., Romão, I. & Zevenhoven, R. CO_2 fixation using magnesium silicate minerals part 1: Process description and performance. *Energy* **41**, 184–191 (2012).
32. Zevenhoven, R., Slotte, M., Åbacka, J. & Highfield, J. A comparison of CO_2 mineral sequestration processes involving a dry or wet carbonation step. *Energy* **117**, 604–611 (2016).
33. Nduagu, E., Bergerson, J. & Zevenhoven, R. Life cycle assessment of CO_2 sequestration in magnesium silicate rock - A comparative study. *Energy Convers. Manag.* **55**, 116–126 (2012).
34. Nduagu, E. *et al.* Production of magnesium hydroxide from magnesium silicate for the purpose of CO_2 mineralisation - Part 1: Application to Finnish serpentinite. *Miner. Eng.* **30**, 75–86 (2012).
35. Hangx, S. J. T. & Spiers, C. J. Coastal spreading of olivine to control atmospheric CO_2 concentrations: A critical analysis of viability. *Int. J. Greenh. Gas Control* **3**, 757–767 (2009).
36. International Institute for Applied Systems Analysis. SSP Database. https://tntcat.iiasa.ac.at/SspDb/dsd?Action=htmlpage&page=40 [last accessed: 15.11.21].
37. Edenhofer, O. *et al. Renewable energy sources and climate change mitigation: Special report of the intergovernmental panel on climate change*. (2012).
38. Green, M. *et al.* Solar cell efficiency tables (version 57). *Prog. Photovoltaics Res. Appl.* **29**, 3–15 (2021).
39. Stehfest, E. et al. *Integrated Assessment of Global Environmental Change with IMAGE 3.0. Model description and policy applications.* (2014).
40. Mazzotti, M. *et al.* Chapter 7. Mineral carbonation and industrial uses of carbon dioxide. In *IPCC Special Report on Carbon dioxide Capture and Storage* 319–338 (2005).
41. Gislason, S. R. & Oelkers, E. H. Carbon storage in basalt. *Science.* **344**, 373–374 (2014).
42. Tang, L., Furushima, Y., Honda, Y., Hasegawa, T. & Itsubo, N. Estimating human health damage factors related to CO_2 emissions by considering updated climate-related relative risks. *Int. J. Life Cycle Assess.* **24**, 1118–1128 (2019).

43. Weidema, B. P. Comparing Three Life Cycle Impact Assessment Methods from an Endpoint Perspective. *J. Ind. Ecol.* **19**, 20–26 (2015).
44. Huijbregts, M.A.J., et al. *ReCiPe 2016 v1.1. A harmonized life cycle impact assessment method at midpoint and endpoint level. Report I: Characterization.* (2017).
45. Federal Reserve Economic Data. U.S./Euro Foreign Exchange Rate. https://fred.stlouisfed.org/series/AEXUSEU [last accessed: 12/02/2021].
46. Federal Reserve Economic Data. Gross Domestic Product: Implicit Price Deflator. https://fred.stlouisfed.org/series/GDPDEF [last accessed: 30.10.21].
47. EUROSTAT. Electricity price statistics. https://ec.europa.eu/eurostat/statistics-explained/index.php?title=Electricity_price_statistics#Electricity_prices_for_non-household_consumers [last accessed: 07.06.2021].
48. Fuss, S. *et al.* Negative emissions - Part 2: Costs, potentials and side effects. *Environ. Res. Lett.* **13**, (2018).
49. Ryberg, M. W., Owsianiak, M., Richardson, K. & Hauschild, M. Z. Development of a life-cycle impact assessment methodology linked to the Planetary Boundaries framework. *Ecol. Indic.* **88**, 250–262 (2018).
50. IPCC. *Climate Change 2013: The Physical Science Basis. Contribution of Working Group I to the Fifth Assessment Report of the Intergovernmental Panel on Climate Change*. (2013).
51. Algunaibet, I. M. *et al.* Powering sustainable development within planetary boundaries. *Energy Environ. Sci.* **12**, 1890–1900 (2019).
52. Bouwman, L. *et al.* Exploring global changes in nitrogen and phosphorus cycles in agriculture induced by livestock production over the 1900-2050 period. *Proc. Natl. Acad. Sci. U. S. A.* **110**, 20882–20887 (2013).
53. De Vries, W., Kros, J., Kroeze, C. & Seitzinger, S. P. Assessing planetary and regional nitrogen boundaries related to food security and adverse environmental impacts. *Curr. Opin. Environ. Sustain.* **5**, 392–402 (2013).
54. Arvidsson, R. *et al.* Environmental Assessment of Emerging Technologies: Recommendations for Prospective LCA. *J. Ind. Ecol.* **22**, 1286–1294 (2018).
55. European Commission - Joint Research Centre - Institute for Environment and Sustainability. *International Reference Life Cycle Data System (ILCD) Handbook - General guide for Life Cycle Assessment - Detailed guidance*. (2010).
56. International Energy Agency. *World Energy Outlook 2019*. (2019).
57. Galán-Martín, Á. *et al.* Sustainability footprints of a renewable carbon transition for the petrochemical sector within planetary boundaries. *One Earth* **4**, 565–583 (2021).
58. Newbold, T. *et al.* Has land use pushed terrestrial biodiversity beyond the planetary boundary? A global assessment. *Science.* **353**, 288–291 (2016).
59. Barnosky, A. D. *et al.* Approaching a state shift in Earth's biosphere. *Nature* **486**, 52–58 (2012).
